# TagBiFC technique allows long-term single-molecule tracking of protein-protein interactions in living cells

**DOI:** 10.1038/s42003-021-01896-7

**Published:** 2021-03-19

**Authors:** Shipeng Shao, Hongchen Zhang, Yong Zeng, Yongliang Li, Chaoying Sun, Yujie Sun

**Affiliations:** 1 State Key Laboratory of Membrane Biology, Biomedical Pioneering Innovation Center (BIOPIC), School of Life Sciences, Peking University, Beijing, China; 2 Beijing Institute of Heart Lung and Blood Vessel Disease, Beijing Anzhen Hospital, Capital Medical University, Beijing, China; 3 The Second Dental Center, Peking University School and Hospital of Stomatology, Beijing, China

**Keywords:** Fluorescence imaging, Cellular imaging

## Abstract

Protein-protein interactions (PPIs) are critical for cellular activity regulation. Visualization of PPIs using bimolecular fluorescence complementation (BiFC) techniques helps to understand how PPIs implement their functions. However, current BiFC is based on fluorescent proteins and the brightness and photostability are suboptimal for single molecule tracking experiments, resulting in either low spatiotemporal resolution or incapability of tracking for extended time course. Here, we developed the TagBiFC technique based on split HaloTag, a self-labeling tag that could conjugate an organic dye molecule and thus offered better brightness and photostability than fluorescent proteins for PPI visualization inside living cells. Through screening and optimization, we demonstrated that the reconstituted HaloTag exhibited higher localization precision and longer tracking length than previous methods. Using TagBiFC, we reveal that the dynamic interactions of transcription factor dimers with chromatin DNA are distinct and closely related to their dimeric states, indicating a general regulatory mechanism for these kinds of transcription factors. In addition, we also demonstrated the advantageous applications of TagBiFC in single nucleosome imaging, light-burden imaging of single mRNA, low background imaging of cellular structures. We believe these superior properties of our TagBiFC system will have broad applications in the studies of single molecule imaging inside living cells.

## Abstract (web summary)

Shao et al. develop an imaging probe, TagBiFC, to visualize protein-protein interaction in living cells with better signal-to-noise ratio and photostability than fluorescent proteins. This probe provides better localization and longer tracking time and authors visualise transcription factor dimerization, single-molecule nucleosome, and background-free mRNA imaging using TagBiFC.

## Introduction

Most cellular functions are executed through protein–protein interactions (PPIs). Compared to the in vitro biochemical assays, studies of PPIs within living cells may not only provide dynamic information that is essential for understanding the relevant functions but also avoid the possibility of unrealistic PPIs as a result of cell lysis and mixing of the contents of different cellular compartments.

A number of methods are available for direct visualization of PPIs in living cells^1^, including bimolecular fluorescence complementation (BiFC)^2^ and fluorescence resonance energy transfer (FRET)^3^. The BiFC approach splits fluorescent proteins (FPs) into two or more fragments, which are unable to fluoresce. When the nonfluorescent fragments are fused with two proteins that can interact with each other, they can complement to form a whole FP and become fluorescent^4–6^. This approach enables visualization and detection of the subcellular location and dynamics of specific protein complexes in the living cellular environment. Recent years have witnessed several important developments of the BiFC techniques. For instance, splitting the stable superfolder GFP between the 10th and 11th β-strands yielded a 17-residue C-terminal peptide GFP11, which is the smallest split FP fragment to date, thereby improving the solubility, maturation time, and complementation efficiency of the system^7^. In addition to detecting PPIs, split FPs (sfGFP1–10/11 and its derivatives) were also evolved for self-assembly and engineered as multimerization scaffold, expanding its applications in targeting recruitment, localization, and imaging^8,9^. BiFC rainbow techniques can achieve live cell visualization of multiple PPIs^10^. Split of photo-switchable (mEos3.2)^11^ and photo-convertible (PAmCherry)^12^ FPs (BiFC-PALM) allows the imaging and tracking of single-molecule PPIs at sub-diffraction resolution in crowded PPI background in living cells. Three-fragment fluorescence complementation coupled with photoactivated localization microscopy allowed nanoscale imaging of ternary complexes^13^. However, despite the exciting progress, current FP-based BiFC is incapable for long-term, precise single-molecule PPI imaging due to their suboptimal brightness and photostability. Compared to FPs, organic dyes are much more bright and photostable, thus are ideal for single-molecule tracking (SMT) in living cells^14^.

Self-labeling tags can conjugate an organic dye molecule to a protein of interest^15^. The self-labeling tags generally utilize microbial enzymes as genetic labels to catalyze the ligation reaction between organic dyes and themselves^16^. One commonly used self-labeling tag is HaloTag, which is derived from the enzyme dehalogenase in *Escherichia coli*. By engineering the dehalogenase, the reaction can stop in the middle of the catalytic cycle and form covalent bonds with the fluorescent substrates, such as the cell membrane-permeable dyes tetramethyl-rhodamine^17^ and Janelia Fluor (JF)^18^. Using HaloTag, single-molecule imaging has been achieved in living cells^19–32^. In contrast, the using of organic dyes for tracking PPIs in living cells has not been realized.

Here, in order to introduce organic dyes into BiFC for precise and long-term tracking of PPIs in living cells, we examined the possibilities of splitting the HaloTag and explored the performance of the split HaloTag system (TagBiFC) for single-molecule PPI imaging in living cells in real time. By inspecting the crystal structure of dehalogenase from *E. coli*^33^, we designed 17 potential split sites for HaloTag, each localizing at the unstructured regions between the neighboring secondary structure units. We successfully screened two split sites at which the complimentary efficiency is significantly higher. Compared with the split photoactivatable fluorescent protein (PAFP) mMaple3, the split HaloTag has better performance in single-molecule localization precision and tracking length. We then investigated the PPIs in living cells using the TagBiFC system. We found both c-Fos and c-Jun can form heterodimers and homodimers with distinct dynamics, which represents a general regulatory mechanism for these kinds of transcription factors (TFs). In addition, our single-molecule imaging and tracking results demonstrate that dimerization can increase both specific and non-specific interaction between dimeric TFs and chromatin DNA. More importantly, the ratio of specific binding increases significantly for dimeric TFs, compared with their monomeric mutants. To expand this approach, here we also used TagBiFC to achieve single nucleosome imaging and explore factors that affect nucleosome dynamics, including transcription and histone variants. Furthermore, we demonstrated background-free and light-burden imaging of single mRNA molecules by TagBiFC. Compared to the FP-based MS2 labeling strategy, TagBiFC is much smaller, and thus can minimize the tagging effect and reveal more realistic life cycle dynamics of mRNA molecules. Lastly, we applied TagBiFC to low background imaging of cellular structure in living cells and achieved long-term tracking of their dynamics. Taken together, these results indicate that TagBiFC can be widely used in imaging and tracking of PPIs and other complicated molecules in living cells with good localization precision and photostability for long-term applications.

## Results

### Rational screening for optimal split sites in HaloTag for the construction of the TagBiFC system

To achieve bimolecular complementary of split HaloTag, the split site should satisfy both of the following criteria. First, the two fragments themselves should not self-associate to from a functional HaloTag molecule with enzymatic activity in the absence of partner proteins. Second, when the two fragments are fused with two proteins that can interact, the interaction between the fusion proteins bring the two split halves to proximity spatially and facilitate the association between the fragments of the split HaloTag, thus generating a self-labeling enzyme that can catalyze the ligation of dye-conjugated substrates to itself.

**Fig. 1:**
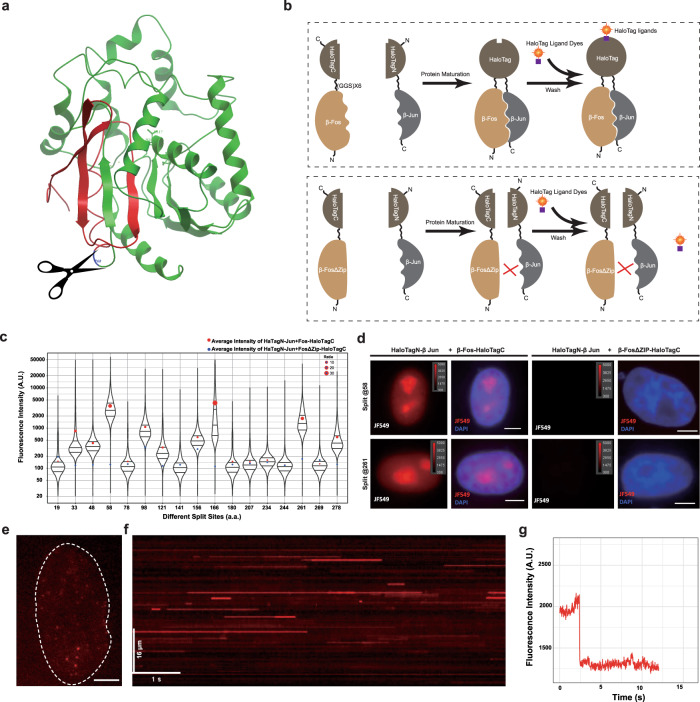
Design and screening for functional site of split HaloTag. **a** Schematic of split HaloTag design. The structure was modified from DhaA (1BN6). The scissor represents the split site and the green and red colors represent the two split halves of HaloTag. The displayed split site is P58 in HaloTag (P68 in 1BN6). **b** Fluorescent retention assay for the screening of potential split sites. Split HaloTag at different positions was fused to two interaction protein fragments, β-Fos and β-Jun, respectively. Functional HaloTag will be reconstituted when β-Fos and β-Jun interact if the split site is proper. Membrane-permeable HaloTag ligand dye was added to the live cells. The fluorescence will remain after washing. Mutation of dimerization domain of β-Fos was used as a control. **c** Violin plot of the retention fluorescence intensity of split HaloTag at different split sites. The red dots are the average intensity of HaTagN-β-Jun+β-Fos-HaloTagC while the blue dots are the average intensity of HaTagN-β-Jun+β-Fos (ΔZip)-HaloTagC. The size of the red dot represents the ratio of HaTagN-β-Jun+β-Fos-HaloTagC to HaTagN-β-Jun+β-Fos (ΔZip)-HaloTagC. **d** Fluorescence images of reconstituted split HaloTag at P58 and G261 split sites. The images were displayed at the same intensity range for comparison. The cell nuclei were stained by DAPI. Scale bars, 5 μm. **e** Typical florescence image of one cell nucleus expressing reconstituted split HaloTag (HaloTag N58-β-Jun and β-Fos-HaloTag C58). The cells were fixed immediately after the incubation of JF549 dye for 15 min. The white dot line delineated the cell nucleus. Scale bar, 2 μm. **f** Kymograph of single-molecule fluorescent trajectory of split HaloTag (from **e**). A long zigzag line was drawn in the nucleus (not shown in **e**) and fluorescence intensity of the line scan was displayed over time. **g** One typical intensity profile showing single-step photobleaching of the TagBiFC signal (from **e**).

Here, based on the crystal structure of dehalogenase (PDB accession code 1BN6)^33^ from *E. coli* and sequence alignments between dehalogenase and HaloTag (Supplementary Fig. 1), we have generated 17 potential split sites (L19, G33, V48, P58, L78, L98, P121, E141, T156, Q166, R180, P207, P234, P244, P261, G269, D278; Fig. 1a and Supplementary Fig. 2). Each of the split sites was in flexible loops between two secondary structure units. The two split fragments were named HaloTagN and HaloTagC and fused with two leucine zipper domains from β-Fos and β-Jun, respectively. Moreover, we also generated a truncation of β-Fos that cannot interact with β-Jun by deleting the zipper sequence within the interaction interface. The coding sequences of these fused fragments HaloTagN-β Jun, β Fos-HaloTagC and β Fos (ΔZIP)-HaloTagC were inserted into the pcDNA3.1(+) plasmid to construct the expression vectors. To avoid the hindrance effect of fused proteins, which may reduce the complementary efficiency, we added a long flexible linker (GGSGGSGGGSGGSGGSGGGS) between the fused proteins and the two halves of split HaloTag.

We then developed a screening method named fluorescent retention assay to identify the functional split sites (Fig. 1b). Briefly, the MDA-MB-231 cells were transfected with interacting and non-interacting proteins pairs. After the maturation of these proteins, HaloTag ligand JF549 was added to the medium. Due to the super-permeability of JF549 (Supplementary Fig. 3a), free diffused dye molecules were depleted from the cells after several times of washing, while the dyes that had been ligated to HaloTag was retained within the cells. Neither HaloTag N nor HaloTag C has residual enzymatic activity after split (Supplementary Fig. 3b, c). Thus, the retention of fluorescent signal in the cells can indicate the complimentary efficiency of each split site. We then used high-throughput fluorescence-activated cell sorting (FACS) to examine the retained fluorescence intensity in individual cells. Among the 17 split sites, several of them demonstrated high complimentary efficiency, including P58, Q166, and P261 (Fig. 1c and Supplementary Fig. 4). However, the uneven distribution of fluorescence intensity of Q166 indicated that the reconstituted HaloTag might form aggregates (Fig. 1c and Supplementary Fig. 4). We thus chose P58 and G261 as the potential and functional split sites of HaloTag in our following experiments.

We next used microscopy to check the fluorescent signals in living cells to verify the complementation of split HaloTag. The HaloTagN-β Jun and β Fos-HaloTagC co-transfected cells yielded a strong fluorescent signal retention in the nucleus, especially in the nucleoli, which is the typical distribution pattern of β-Jun and β-Fos interaction pairs^34^. In contrast, the fluorescent intensity was extremely low in HaloTagN-β-Jun and β-Fos (ΔZip)-HaloTagC co-transfected cells (Fig. 1d). Taken together, these results indicate that the split HaloTag at positions P58 and P261 can complement upon fusion to interacting protein pairs in living cells. We then used TagBiFC at P58 for all the subsequent experiments unless otherwise indicated.

### Characterization of the TagBiFC system

We first proved that we can achieve single-molecule sensitivity for detection of PPIs in living cells by the split HaloTag system (JF549). Using the highly inclined and laminated optical sheet (HILO) illumination mode, we were able to clearly observe single fluorescent puncta in the nucleus in fixed HeLa cells (Fig. 1e). Kymograph and photobleaching analyses confirmed that the single puncta correspond to single-molecule signal (Fig. 1f, g and Supplementary Fig. 5). These results indicate that the reconstituted split HaloTag molecules retain the single-molecule properties as the intact HaloTag, and split HaloTag can detect PPIs in fixed cells at the single-molecule level.

**Fig. 2:**
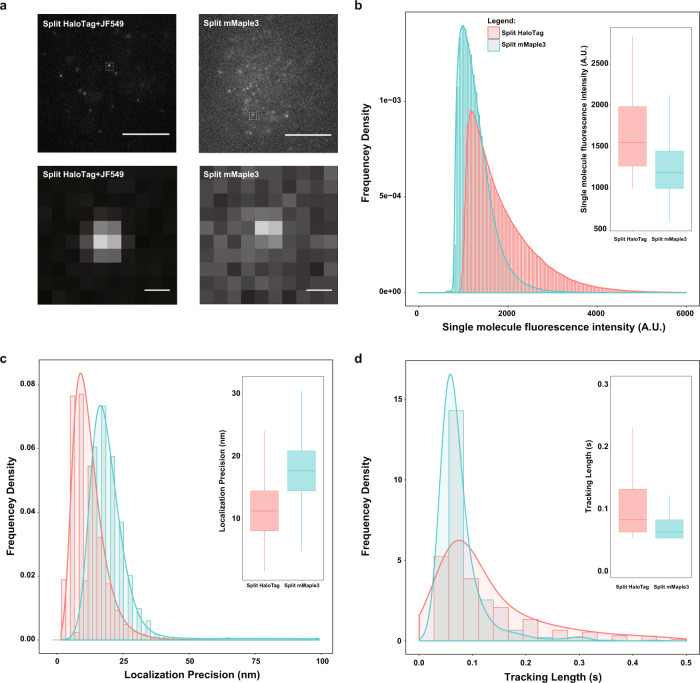
Comparison of single-molecule localization precision of split HaloTag and split mMaple3. **a** Single-molecule fluorescence images of split HaloTag and split mMaple3 in living cells. Split HaloTag (JF549) and split mMaple3 were fused with β-Fos and β-Jun (β-Fos-HaloTag C58/mMaple3 C173, HaloTag N58/mMaple3 N173-β-Jun) and co-expressed in MDA-MB-231 cells. The upper panel: whole-cell images, scale bars: 5 μm; the lower panel: single-molecule zoom-in images of the white boxes in the upper panel, scale bars: 300 nm. **b** Histogram of single-molecule intensity of split HaloTag and split mMaple3 in living cells. Inset is the boxplot of the same data. **c** Histogram of single-molecule localization precision of split HaloTag and split mMaple3 in living cells. Inset is the boxplot of the same data. **d** Histogram of single-molecule tracking length of split HaloTag and split mMaple3 in living cells. Both the BiFC-Tag and split mMaple3 were fused with histone H2A and H3. Inset is the boxplot of the same data.

We then directly compared the performance of imaging PPIs in living cell by our TagBiFC system and the FP-based BiFC system. We chose mMaple3 as a comparison control because it is a widely used PAFP in SMT and localization-based super-resolution imaging^35^. Based on our previous knowledge of split mEso3.2^11^, we split mMaple3 at K173 and fused mMaple3N and mMaple3C with β Jun and β Fos, respectively. Expression of split HaloTag and split mMaple3 in living cells and using the HILO illumination mode, single-molecule signals can be observed in living cell nucleus (Fig. 2a). These single-molecule images indicated that the brightness of reconstituted split HaloTag (JF549) is higher than that of split mMaple3 (Fig. 2b) under the same imaging conditions. As the brightness of single-molecule fluorescent probes directly affects the localization precision, the reconstituted split HaloTag (JF549) showed nearly twofold improvement in localization precision compared with split mMaple3 (Fig. 2c and Supplementary Fig. 6). In single-molecule live cell imaging, molecules keep moving around and tracking them requires short exposure time^36^. Thus, the brighter probes (i.e., JF549) used in TagBiFC can emit more photons during the short exposure period and is suitable for imaging and tracking PPIs in living cells.

We next characterized the photostability of TagBiFC. In order to rule out the possibility that disappearing of the single-molecule signal is a result of disassociation from the binding site and moving away from the imaging focal plane, we used BiFC of histone proteins here because they are stably bound to chromatin. To do so, we expressed HaloTagN-H2A+H3-HaloTagC and mMaple3N-H2A+H3-mMaple3C in MDA-MB-231 cells. We measured the time length of each individual nucleosome. The data indicated that the tracking length of TagBiFC (JF549) is much longer than that of split mMaple3 (Fig. 2d). The longer tracking length contains more information about the dynamic details of labeled molecules. We also compared the stability of split HaloTag with intact HaloTag by tracking their decay process using western blot. The similar decay process indicated that splitting HaloTag does not influence its degradation dynamics (Supplementary Fig. 7). Overall, the characterization demonstrates the superior performance of TagBiFC in tracking PPIs at the nanometer scale for extended time course in living cells than other existing methods.

We also measured the maturation time of TagBiFC using the inducible interaction protein pairs FKBP and FRB fused with HaloTagN and HaloTagC, respectively^37^ (Supplementary Fig. 8). The results indicated that the half maturation time of split HaloTag is about 5 h (Supplementary Fig. 8d), longer than most FP-based BiFC^11,12^. Nevertheless, for SMT experiments, one important labeling criterion is sparse labeling^38^ so that multiple emitters would rarely localize within a diffraction-limited region. Therefore, it is practically beneficial to have only a small portion of labeled molecules being mature during the imaging process.

### Dissecting the dynamic interaction of c-Fos and c-Jun with chromatin in living cells using TagBiFC

The dimeric AP-1 TF complexes have been proven to play critical roles in controlling cell proliferation and differentiation by regulating gene expression in response to various stimuli^39^. Multiple AP-1 subunits are expressed at the same time, including but not restricted to c-Fos, c-Jun, FosB, JunB, JunD, Fra1, Fra2, ATF2, ATFa, and ATF3^40^. Each member of the AP-1 family TFs can interact with each other to form heterodimers, which have distinct roles in regulating the transcription of target genes. In addition, some of them can form homodimers, thus creating a high heterogeneity in their dimer composition^41^. Analyzing the sequences of AP-1 family TFs has uncovered that they all belong to the family of bZip proteins and share the same dimerization and DNA-binding domains while their transcription activation domains vary^42^. The DNA-binding and transcription activation domains of the AP-1 family can function as independent modules. The DNA-binding domains use a surface to contact with DNA, whereas the leucine zipper domains promote the formation of homodimers or heterodimers with other bZip proteins^42^. Thus, the functional activity of AP-1 in any given cell at any moment depends on their dimeric states. This diversity of AP-1 components has complicated our understanding of the relationship of different AP-1 composition and function, especially the dynamic interaction process with chromatin. In fact, although full-length HaloTag has been fused to c-Fos or c-Jun to visualize the dynamics of AP-1 TFs, it is unclear whether the molecules under observation are monomer, homodimer, or heterodimer. Here we used TagBiFC to investigate specific AP-1 homodimers and heterodimers and dissect their differences in interaction with the chromatin.

We fused c-Fos and c-Jun with full-length HaloTag, split HaloTagN, and HaloTagC, which generated 6 fusion constructs, i.e. c-Fos-HaloTag, c-Jun-HaloTag, c-Fos-HaloTagC, c-Jun-HaloTagC, HaloTagN-c-Fos, and HaloTagN-c-Jun. We first checked whether the fusion of split HaloTag affected the location and dimerization of c-Fos and c-Jun by visualizing the distribution of c-Fos-HaloTag, c-Jun-HaloTag, and c-Fos-HaloTagC+HaloTagN-c-Jun. The results indicated that the fusion influenced neither the localization nor the capability to form dimers of c-Fos and c-Jun. Furthermore, by co-expressing c-Fos-HaloTagC with HaloTagN-c-Fos or c-Jun-HaloTagC with HaloTagN-c-Jun, we found both c-Fos and c-Jun can form homodimers (Supplementary Fig. 9a). Interestingly, the c-Fos homodimers mainly localized in the cytoplasm while c-Jun homodimers exclusively remained in the nucleus (Supplementary Fig. 9a, b).

**Fig. 3:**
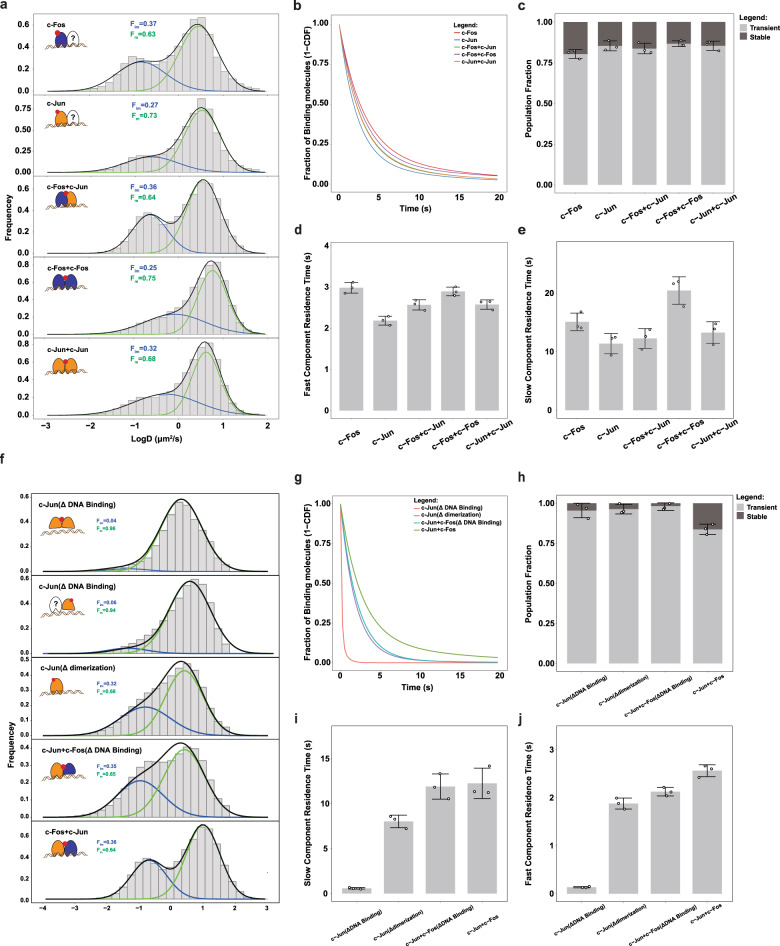
Dynamic interaction of c-Fos and c-Jun with chromatin in living cells. **a** Normalized histograms of the log diffusion coefficient for c-Fos-HaloTag (*N* = 10 cells, *n* = 1,610,629 trajectories), c-Jun-HaloTag (*N* = 11 cells, *n* = 179,948 trajectories), c-Fos-HaloTag C58+HaloTag N58-c-Jun (*N* = 10 cells, *n* = 737,531 trajectories), c-Fos-HaloTag C58+HaloTag N58-c-Fos (*N* = 10 cells, *n* = 2,088,228 trajectories), and c-Jun-HaloTag C58+HaloTag N58-c-Jun (*N* = 10 cells, *n* = 1,316,262 trajectories) in living MDA-MB-231 cells. All the histograms were fitted with a two-component Gaussian function. The blue color lines indicate the fraction of proteins in the chromatin-bound (both specific and non-specific) state while the green color lines indicate the fraction of proteins in fast diffusion state. The black lines are the sum of the two Gaussian functions. **b** Cumulative frequency distribution of the dwell times for c-Fos (*N* = 12 cells, *n* = 10,618 trajectories), c-Jun (*N* = 10 cells, *n* = 8452 trajectories), c-Fos+c-Jun (*N* = 10 cells, *n* = 44,243 trajectories), c-Fos+c-Fos (*N* = 10 cells, *n* = 1538 trajectories), and c-Jun+c-Jun (*N* = 10 cells, *n* = 10,271 trajectories) in living MDA-MB-231 cells. All the histograms were fitted with a two-component exponential decay function. **c** Fraction of stable and transient binding molecules of different c-Fos and c-Jun combinations. Error bars represent standard deviation from double exponential fitting. **d**, **e** Residence time of transient (**d**) and stable (**e**) binding molecules of different c-Fos and c-Jun combinations. Error bars represent standard deviation from double exponential fitting. **f** Normalized histograms of the log diffusion coefficient for c-Jun (ΔDNA binding)-HaloTag C58+ HaloTag N58-c-Jun (ΔDNA binding) (*N* = 10 cells, *n* = 90,436 trajectories), c-Jun (ΔDNA binding)-HaloTag (*N* = 10 cells, *n* = 85,150 trajectories), c-Jun (ΔDimerization)-HaloTag (*N* = 10 cells, *n* = 91,165 trajectories), c-Fos (ΔDNA binding)-HaloTag C58+HaloTag N58-c-Jun (*N* = 10 cells, *n *= 69,008 trajectories), and c-Fos-HaloTag C58+HaloTag N58-c-Jun (*N* = 10 cells, *n* = 1,316,262 trajectories) in living MDA-MB-231 cells. **g** Cumulative frequency distribution of the dwell times for c-Jun (ΔDNA binding) (*N* = 10 cells, *n* = 1489 trajectories), c-Jun (ΔDimerization) (*N* = 10 cells, *n* = 2067 trajectories), c-Fos (ΔDNA binding)+c-Jun (*N* = 10 cells, *n* = 9702 trajectories), and c-Fos+c-Jun (*N* = 10 cells, *n* = 10271 trajectories) in living MDA-MB-231 cells. **h** Fraction of stable and transient binding molecules of different c-Fos and c-Jun mutants. Error bars represent standard deviation from double exponential fitting. **i**, **j** Residence time of transient (**i**) and stable (**j**) binding molecules of different c-Fos and c-Jun mutants. Error bars represent standard deviation from double exponential fitting.

To quantify the dynamics of different c-Fos and c-Jun dimers, we imaged the cells continually using 10 ms exposure time to capture the dynamics of all labeled molecules. The mean square displacement analysis revealed that the single-molecule motion behaviors vary among different compositions of c-Fos and c-Jun dimers (Supplementary Fig. 9c). For c-Fos homodimers that distributed both in the cytosol and nucleus, we found that the molecules in the cytosol moved faster and more freely than that in the nucleus (Supplementary Fig. 9d). We then calculated the diffusion coefficient of the five catalogs of molecules in the nucleus. Since the histogram revealed two obvious populations, we used two-component Gaussian function to fit the distribution with the slow component assigned as the chromatin-bound population, whose slow motion reflects the chromosomal dynamics, and the fast component assigned as the fast diffusion population, whose motion reflects freely diffusing molecules (Fig. 3a). The data show that, while majority of TF dimer molecules were mobile and only a small fraction was stationary, the proportions varied for different kinds of dimers (Fig. 3a). The chromatin-binding fraction of c-Fos (37%) is higher than that of c-Jun (27%). The chromatin-binding fraction of homodimer (c-Fos and c-Fos, c-Jun and c-Jun) and heterodimer (c-Fos and c-Jun) is in between (Supplementary Fig. 10). We further revealed that the expression level of the TagBiFC fusion proteins was similar to the endogenous ones (Supplementary Fig. 11), thus ruling out the possibility that overexpression of TagBiFC-tagged TFs might affect their chromatin-binding fraction.

Next, to discriminate transient and stable binding events of c-Fos and c-Jun dimers, we used an alternative imaging strategy to image only the chromatin-binding fraction. Owing to the photobleaching of fluorescent probes, both photobleaching and dissociation contribute to the loss of the fluorescent signal. In order to minimize the effect of photobleaching on the calculation of residence time, we performed time-lapse illumination with a 10-ms camera integration time interspersed with a dark period of 490 ms. We then used a double-exponential decay function, corresponding to specific and non-specific DNA binding, to fit the survival curves of c-Fos and c-Jun dimers (Supplementary Fig. 12). After correcting for photobleaching, we estimated average residence times for c-Fos and c-Jun dimers (Fig. 3b). The stable binding fraction was similar (~20%) among all these combinations (Fig. 3c). Moreover, the fast component residence time of c-Fos was higher than that of c-Jun, while that of heterodimer and homodimers were in between (Fig. 3d). The slow component residence time of c-Fos was also higher than that of c-Jun, while that of c-Fos+c-Jun heterodimer and c-Jun+c-Jun homodimer were in between. However, the homodimer of c-Fos, which rarely localized in the nucleus, bound chromatin stably with the longest residence time (Fig. 3e and Supplementary Fig. 13). Taken together, these data demonstrated that the same TF molecule may form dimers with different partners, and different combination of c-Fos and c-Jun TF dimers have distinct interaction dynamic behaviors with chromatin, which may represent a general regulatory mechanism for the dimeric TFs. The TagBiFC system is extremely suitable and powerful for imaging- and tracking-specific TF homodimers and heterodimers.

### Dimerization promotes the long-term interaction between TFs with chromatin

After we have revealed the distinct dynamic of different c-Fos and c-Jun dimers, we next wanted to investigate the role of dimerization in controlling the interaction between dimeric TFs and chromatin in living cells. We created different mutative constructs of c-Fos and c-Jun that cannot bind to chromatin (ΔDNA binding) or dimerization (ΔDNA dimerization)^43^.

We first tracked the dynamics of a c-Jun mutant incapable of binding chromatin. The results indicated that DNA-binding domain is critical for the dimeric TF binding to chromatin. However, we also observed a small fraction of c-Jun (ΔDNA binding) bound to chromatin. We speculate that this fraction corresponds to c-Jun (ΔDNA binding) dimerizing with other endogenous AP-1 family members with chromatin-binding capability (Fig. 3f and Supplementary Fig. 14). We then tracked a c-Jun mutant incapable of dimerization with other AP-1 family members. The chromatin-binding fraction of c-Jun (ΔDNA dimerization) was similar with that of c-Fos+c-Jun dimer. In addition, we also used a c-Fos mutant that was incapable of chromatin binding yet was capable of dimerization with other AP-1 TFs. We forced this c-Fos mutant to form dimer with wild-type c-Jun via the complementary of split HaloTag. We also found that the dimer with only one DNA-binding domain had similar chromatin-binding fraction with the wild-type dimers. These results revealed that dimerization had minimal effect on the chromatin binding of dimeric TFs.

**Fig. 4:**
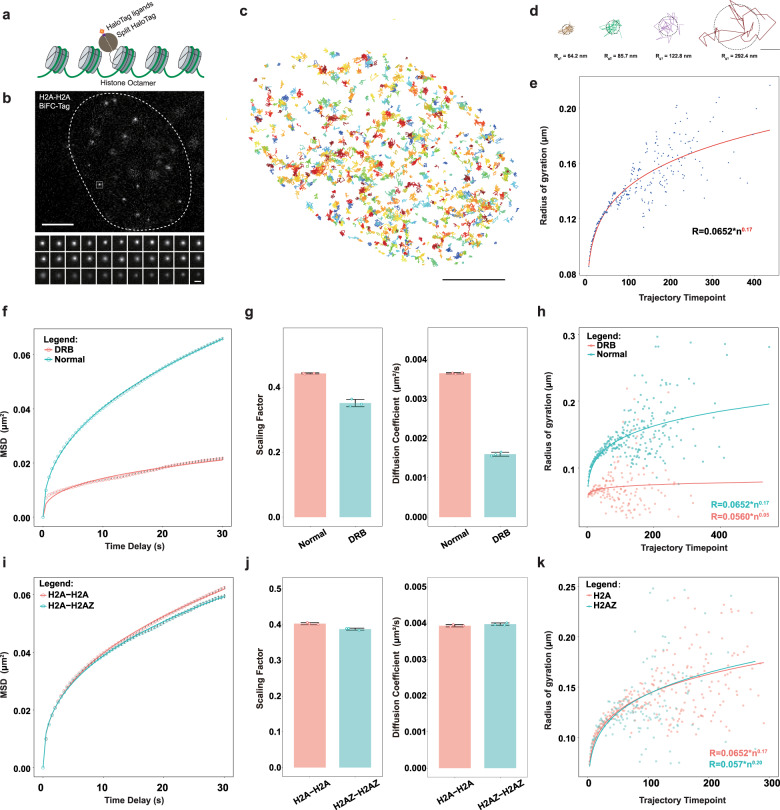
Single nucleosome imaging using TagBiFC in living cells. **a** Schematic of single nucleosome imaging method by Tag BiFC. Two components of histone octamers are fused with BiFC-Tag (H2A-HaloTag C58+HaloTag N58-H2A) and can be reconstituted as an intact nucleosome in living cells. **b** Representative fluorescence image of single nucleosome labeling using TagBiFC. The outline of the nucleus is drawn out in white line. A zoom snapshot of a single nucleosome is shown in the bottom. The exposure time is 200 ms and interval between each frame is 500 ms. Scale bar, 5 μm (top) and 1 μm (bottom). **c** Single-molecule tracking trajectory of nucleosome in a typical nucleus. Scale bar, 5 μm. **d** Heterogeneity of single nucleosome motion within the same cell nucleus. Trajectory and radius of gyration of four typical single nucleosomes are shown. Scale bar, 100 nm. **e** Radius of gyration of all the nucleosomes as a function of trajectory length. The data were fitted with power law. **f**–**h** Mean square displacement (**f**), scaling factor, diffusion coefficients (**g**), and radius of gyration (**h**) of all the nucleosomes under normal or DRB-treated condition. Data are displayed as mean ± standard deviation. **i**–**k** Mean square displacement (**i**), scaling factor, diffusion coefficients (**j**), and radius of gyration (**k**) of all the nucleosomes of H2A-HaloTag C58+HaloTag N58-H2A and H2A-HaloTag C58+HaloTag N58-H2AZ. Data are displayed as mean ± standard deviation.

We next calculated the residence time of c-Jun mutants by time-lapse illumination. The survival probability curve of c-Jun (ΔDNA binding) decayed to zero quickly, indicating that DNA-binding domain is most important for TFs to contact with DNA for a long time (Fig. 3g and Supplementary Fig. 15). Interestingly, we also found that the fraction of slow residence time of c-Fos+c-Jun was dramatically higher than all the c-Jun mutants (Fig. 3h). Moreover, both the fast and slow residence time of c-Jun (ΔDimerization) and c-Jun+c-Fos (ΔDimerization) were significantly lower than that of c-Fos+c-Jun (Fig. 4i–j and Supplementary Figs. 16 and 17). The residence time analysis results suggest that dimerization contribute greatly for the dimeric TFs to stay at the targeted sequence for longer time. Taken together, these results indicate that, although the DNA-binding fraction is not influenced by the dimerization process, the dimerization contributes to the long residence time on chromatin, which is crucial for the role of TFs in transcription.

### TagBiFC allows single nucleosome imaging in living cells

The dynamic behavior of nucleosomes plays an important role in DNA replication, transcription, and damage repair in eukaryotic organisms. A number of factors, such as DNA methylation, histone modification, and histone variants have important regulatory effects on the dynamic characteristics of nucleosomes and chromatin. Given the single-molecule sensitivity and composition-specific detection capability, it is possible to use TagBiFC to study the dynamics of individual nucleosomes composed of different histone variants in the octameric complex.

We first measured the general dynamics of nucleosomes in HeLa cells by fusing histone H2A with HaloTagN and HaloTagC, respectively (Fig. 4a). The single nucleosome molecules were of high signal-to-background ratio and could be tracked for a long period (Fig. 4b). The single-molecule trajectories (with same trajectory length) (Fig. 4c) showed large heterogeneity (Fig. 4d). We then classified them according to the length of the trajectory and plotted a relationship between the radius of gyration and the length of the trajectory. We found that, within a shorter trajectory length, the radius of gyration increases rapidly with the increase of the length of the trajectory (Fig. 4e). These data suggest that accurate measurements of confined diffusion with a large radius of gyration requires a long duration of trajectory recording, indicating the importance of photostability of TagBiFC.

Many reasons contribute to the heterogeneity of nucleosome movement, such as transcription, the radial positions in the nucleus, epigenetic states, different cell cycle stages, etc. We next explored what these effects may regulate the motion of single nucleosome. We used DRB to inhibit RNA Pol II elongation for 10 h and then tracked single nucleosome movements. All parameters including the mean square displacement, scaling factor, diffusion coefficients, and radius of gyration decreased dramatically in the transcription-inhibited state (Fig. 4f–h). These data indicate that inhibition of transcription leads to a condensed state of chromatin with low nucleosome mobility and transcription contributes greatly to chromatin structure and dynamics.

Next, we used TagBiFC to study how histone composition affects the nucleosome dynamics. As a brief demonstration, we compared the single nucleosome dynamics of core histones (H2A–H2A) with H2A.Z variant histones (H2A–H2A.Z). The results indicated nearly identical motion dynamics of H2A.Z-composed nucleosomes and the core histone-composed nucleosomes (Fig. 4i–k).

### Light-burden imaging of single mRNA molecules in live cells using TagBiFC

**Fig. 5:**
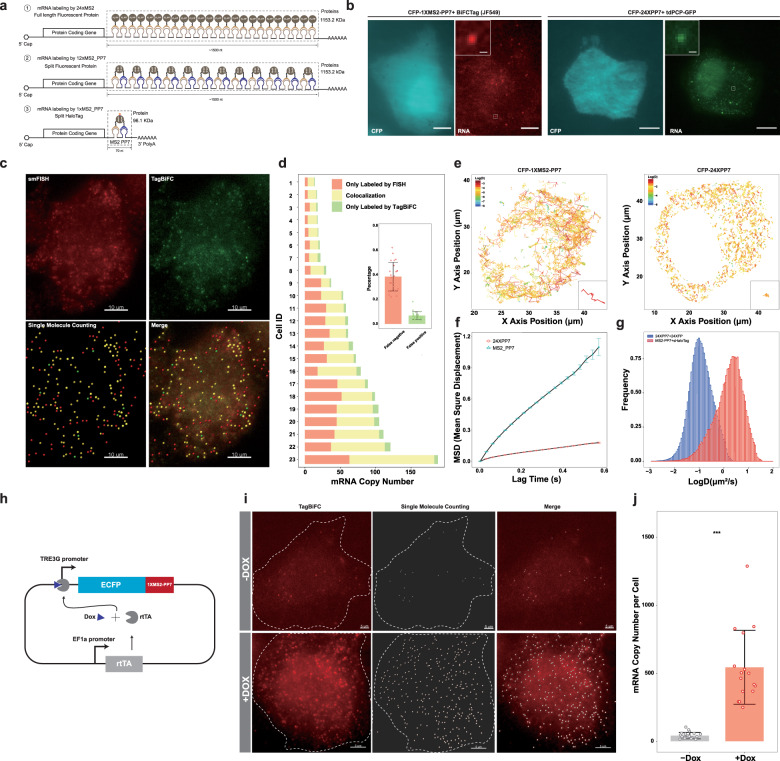
Burden-lighter imaging of single mRNA and cirRNA molecules in live cells using TagBiFC. **a** Schematic comparation of three single-molecule imaging methods of mRNAs in living cells. ① mRNA labeling using 24×MS2 hairpin repeats and full-length fluorescent protein fusion with tdMCP (or MCP). ② mRNA labeling using 12×MS2_PP7 hybrid hairpin repeats and split fluorescent protein fusion with MCP and PCP. ③ mRNA labeling using 1×MS2_PP7 hybrid hairpin repeat and BiFC-Tag fusion with MCP and PCP. The estimation of the labeling tag length and binding protein mass are shown. **b** Representative fluorescence images of single CFP mRNA labeling using 1×MS2-PP7/TagBiFC (left) or 24×PP7 (right). The 1×MS2-PP7 or 24×PP7 sequence was inserted in the 3’ UTR of coding sequence of CFP. tdPCP-HaloTag C58+HaloTag N58-tdMCP or tdPCP-EGFP was transfected to label the mRNA. Inset shows single-molecule signal from a high magnification of the box region. Scale bar, 5 μm. Inset scale bar, 1 μm. **c** Representative fluorescence images of single CFP mRNA 3D projection labeled using TagBiFC and single-molecule FISH. The mRNAs were counted using the Spots function of Imaris. Scale bar, 5 μm. **d** Quantification of the colocalization of TagBiFC and FISH signal in multiple cells. Inset shows the false-positive and false-negative ratio of TagBiFC for mRNA labeling. **e** Single-molecule tracking trajectory of CFP mRNA labeled by 1×MS2-PP7 or 24×PP7. Insets show single-molecule trajectories for a high magnification. **f** Mean square displacement of CFP mRNA labeled by 1×MS2-PP7 or 24×PP7. Data are displayed as mean ± standard deviation. **g** Histogram of diffusion coefficient of CFP mRNA labeled by 1×MS2-PP7 or 24×PP7. **h** Schematic of plasmid used for inducible reporter mRNA expression. Two expression cascades were cloned into one plasmid to ensure the co-appearance in the same cells. The rtTA was driven by EF1a promoter while CFP-MS2-PP7 was driven by tetracycline responsible element (TRE) promoter. **i** Representative fluorescence images of single CFP mRNA 3D projection labeled using TagBiFC in the absence or presence of 10 μg/mL tetracycline. The mRNAs were counted using the Spots function of Imaris. Scale bar, 5 μm. **j** CFP mRNA copy number per cell in the absence or presence of tetracycline. ***Statistically significant difference (*p* value = 2.193e−06, paired *t* test, *n* = 16 for the +Dox group and *n* = 23 for the −Dox group).

Besides tracking PPIs, with the high brightness of organic dyes and low fluorescence background of TagBiFC, it is possible to use TagBiFC with minimal tagging load, i.e., one HaloTag, to achieve single-molecule imaging of biological macromolecules, which otherwise requires multiple tandem repeats when using FPs as the probe. To this end, we compared TagBiFC and FP-based BiFC for mRNA imaging. In the past two decades, most of the SMT experiments of mRNA in various model organisms rely on the genetic encoded RNA tag MS2^44^ and PP7^45^ (Fig. 5a①). This approach requires MCP/PCP expression in excess, which results in a high background. Alternatively, MS2-PP7 hybrid aptamer coupled with split FPs has been demonstrated to enable background-free imaging of mRNA at the single-molecule level^46^ (Fig. 5a②). However, in order to gain sufficient signal-to-background ratio for mRNA single-molecule imaging, both methods require multiple copies of these RNA aptamers in tandem repeat to label the target mRNA, adding high load to the mRNA molecule and thus resulting in underestimation of the mRNA motion dynamics. Furthermore, the long tag and multiple coat protein binding may alter the intrinsic turnover and transportation processes of the labeled mRNA molecules.

We sought to harness the TagBiFC system to label single mRNA molecule in living cells (Fig. 5a③). We reason that TagBiFC can achieve background-free imaging, since only the coat proteins bound to the mRNA molecules are fluorescent and the excess unbound coat proteins are non-fluorescent. Moreover, as we have proved that the brightness and photostability of TagBiFC is much higher than the split FPs, only 1 copy of MS2-PP7 hybrid aptamer can give enough signal-to-background ratio for single-molecule imaging of mRNA with light burden. We used cyan fluorescent protein (CFP) mRNA as a reporter to compare the performance of mRNA single-molecule labeling and tracking by TagBiFC and the conventional method. 24×PP7 and 1×MS2-PP7 were inserted in the 3’ end of the CFP-coding gene. tdPCP-GFP or tdMCP-HaloTagN and tdPCP-HaloTagC were co-expressed to label CFP mRNA. Similar single CFP mRNA molecules could be detected in the corresponding cells (Fig. 5b). In addition, no fluorescent puncta could be monitored in the absence of mRNA molecules (Supplementary Fig. 18). To direct compare the false-positive or false-negative rate of TagBiFC for mRNA imaging, we analyzed the colocalization of CFP-MS2-PP7 reporter mRNA labeled using single-molecule FISH (smFISH) and TagBiFC (Fig. 5c, d). The false-positive rate of TagBiFC, which has TagBiFC signal but not FISH signal, is quite low (~5%), indicating the robustness and reliability of TagBiFC for interacting protein labeling and background-free mRNA imaging. However, the false-negative rate, which corresponds to RNA molecule that only labeled by FISH, is about 40%. Taken together, these results indicate that TagBiFC performs as robust as the traditional method.

Importantly, the single-molecule trajectories demonstrated very different patterns for the two labeling methods (Fig. 5e). TagBiFC-labeled mRNAs showed more straight and persistent trajectories than 24×PP7-labeled mRNAs, which were more confined and less mobile. The mean square displacement (Fig. 5f) and diffusion coefficient (Fig. 5g) showed that TagBiFC-labeled mRNAs diffused about ten times faster than 24×PP7-labeled mRNAs, suggesting that the tag size can drastically influence the dynamic behavior of mRNA molecules. Therefore, the small tag size and bright signal would allow TagBiFC with broad applications in RNA imaging experiments.

We then use TagBiFC to image and count gene expression of upregulation and downregulation. We used a tetracycline-inducible system to image the CFP-1×MS2-PP7 reporter gene expression in the presence or absence of tetracycline (Fig. 5h). Without tetracycline, the leaky expression of tetracycline responsible element promoter generated about 40 single mRNA molecules per cell. After the induction using 10 μg/mL Doxycycline for 2 h, about 500 mRNA molecules were transcribed (Fig. 5i, j), indicating the capability of TagBiFC for absolute mRNA counting.

### Low background imaging of cellular structure in live cells using TagBiFC

Lastly, we used the TagBiFC system to achieve low background imaging of cellular structures. Because the TagBiFC technique is only fluorescent in the dimer form, either through direct PPIs or binding to adjacent structural unit, we reasoned that TagBiFC would be advantageous in labeling and imaging of periodic cellular structures.

**Fig. 6:**
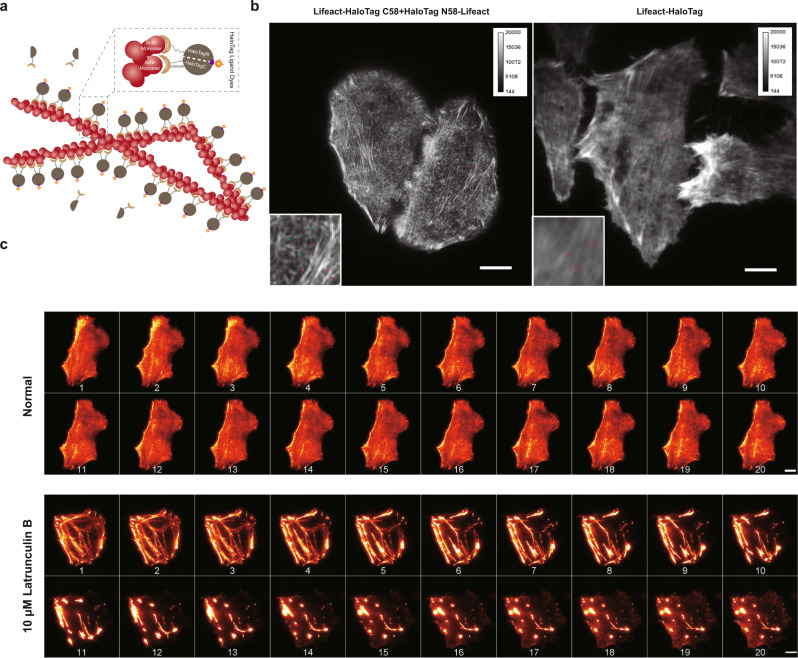
Background-free imaging of actin filaments in live cells using TagBiFC. **a** Schematic of actin filament imaging using BiFC-Tag. The two split halves of HaloTag were both fused with F-actin-binding peptide lifeact (Lifeact-HaloTag C58+HaloTag N58-Lifeact). Only two halves that bind to two adjacent actin units of the filaments and reconstitute an intact HaloTag can be imaged under microscopy. **b** Comparation of F-actin labeling results by BiFC-Tag (left) and HaloTag (right). Insets show the box regions in the images. Red arrow heads indicate the thick stress fibers that can be labeled using both methods while the green arrow heads indicate the thin filaments that can only be labeled using TagBiFC. Scale bars: 5 μm. **c** BiFC-Tag allows long-term imaging of F-actin dynamics under normal or Latrunculin treatment conditions. Representative snapshots were displayed, and time post treatment is shown under each snapshot. Scale bars: 5 μm.

Here we used TagBiFC to achieve low background imaging of F-actin in living cells with long-term tracking capability. We fused the two halves of split HaloTag to the F-actin-binding peptide Lifeact. Lifeact is a 17-amino acid peptide, which stained filamentous actin in eukaryotic cells without interfering with actin dynamics^47^. Only the two halves that bind to adjacent actin monomer in F-actin filaments are fluorescent while the overexpressed probes diffusing within the cells are invisible (Fig. 6a and Supplementary Fig. 19). Compared with the full-length HaloTag fusion control cells in which only thick actin filaments (mainly stress fiber) were emerged from the background, thin actin filaments could be clearly resolved in the TagBiFC-labeled cells (Fig. 6b). We also demonstrated that TagBiFC-labeled F-actin can be monitored at a long-time scale both in normal or drug-treated condition (Fig. 6c, d). Taken together, our TagBiFC system shows great performance in labeling and imaging of periodic cellular structures.

## Discussion

Revealing the formation, distribution, and dynamics of PPIs in situ in living cells can deepen our understanding of how cells coordinate their multiple components to fulfill their activities. Here we have developed a novel method TagBiFC to image and track PPIs, mRNA, and actin filaments in living cells at a single-molecule level in real time. Compared with the other methods, TagBiFC is superior in the following aspects. First, TagBiFC can provide higher spatial and temporal resolution, enabling accurate localization and long-term tracking of PPIs. In this study, we have achieved about 10 nm localization accuracy and 10 ms temporal resolution. In addition, recent study has uncovered that, using low concentration of dissolved oxygen with a reducing-plus-oxidizing system, photobleaching and photoblinking of organic dyes can be strongly suppressed, which enabled SMT for super-long time scale^48^. Second, a much broader spectrum range of organic dyes are now available than PAFPs. Several recently developed JFs have formed a palette with excitation and emission ranging from blue to the far-red^18,49,50^. The versatility of these new dyes can give us more flexibility for single-molecule imaging of PPIs in living cells. Third, some PAFPs generally exhibit a certain degree of dimerization, which may perturb the localization and dynamics of target proteins^35^. Meanwhile, no reports about dimerization of self-labeling tags have been reported. Therefore, the small tag size and high brightness make TagBiFC a suitable single-molecule imaging tag for the target molecules whose dynamics and turnover are sensitive to the tag size.

We have taken advantage of TagBiFC to investigate the dynamic interaction process of dimeric TFs (c-Fos and c-Jun) with chromatin. We have found that both c-Fos and c-Jun can form homodimer and heterodimer in living cells with distinct dynamics. We have proved that the partnership plays a critical role in controlling and regulating the dimeric TF binding to chromatin. Furthermore, we also found that the distinct dimers reside on chromatin at different time points. For TFs, long resident time on chromatin might contribute to transcriptional output. More importantly, we have demonstrated that dimerization of these TFs does not affect the total chromatin-binding fraction, instead increase the stable binding fraction and residence time. The mechanism we revealed here may represent a general rule for the regulation of dimeric TF interaction with the chromatin. In the future, it is of great interest to see whether other dimeric TFs also follow this rule. Additionally, in order to eliminate the influence of differential overexpression of TagBiFC-labeled proteins on chromatin binding, it is more accurate to perform genome editing to insert TagBiFC to the endogenous locus in the future.

In addition to detection of PPIs, we also expanded TagBiFC for the labeling of other cellular molecules and structures. First, we fused histone proteins with TagBiFC and investigated single nucleosome dynamics in living cells. We think that this would be useful for tracking nucleosomes that are composed of different histone variants. Second, using TagBiFC to label and track mRNA molecules in living cells has minimal effect on the mRNA dynamics. Up to now, transient transfection of plasmids remains the mainstream in most of the mRNA labeling experiments in living cells. Using TagBIFC, we can easily achieve long-term single-molecule imaging of mRNA with only 1×MS2-PP7. With the smaller tag size, direct labeling of the endogenous RNA using CRISPR-based genome-editing technique might be achieved easily in the future. As for the 40% false-negative detection rate of TagBiFC in mRNA imaging, we speculate that this is due to the conformation requirement of the split two halves to reconstitute an intact HaloTag on a flexible RNA molecule. This has no influence on the imaging and tracking of single-molecule RNA. In most of the circumstances, we are more interested in dissecting the dynamics of single-molecule mRNA in living cells when using TagBiFC. As for the counting of copy number of single RNA molecule, we can divide the detection count by the detection coefficient (about 0.6) for the accuracy. Lastly, we used TagBiFC to label F-actin in living cells with much repressed background. We think other cellular structures can also be labeled by TagBiFC, including microtubule, intermediate filaments, and lamins.

The functionality of TagBiFC can be further expanded in the future. On one hand, the cleavage sites used to construct the split HaloTag system also provide a good reference for the development of other split self-labeling tags, such as SnapTag^51^, CLIPtag^52^, and TMPTag^53^. These tag proteins can also be split in the similar manner to expand the multi-color capability for detection of multiple PPIs in a single cell. On the other hand, the HaloTag might be split into three fragments like mIrisPF^13^, making it a suitable tool for imaging and tracking of three-component complex at the single-molecule level in living cells. Therefore, we expect a broad applicability for the split HaloTag system. The limitation of the split HaloTag system is the irreversible process of the reconstituted HaloTag, which is also the major problem of most BiFC systems. Due to fact that the dye labeling and imaging processes are temporally separated, we could not rule out the possibility that the labeled protein might have dissociated from the protein partner with which it was bound during labeling. TagBiFC might be limited to image stable complexes that remain associated for much longer time than the time it takes to label and then image. Further efforts should be dedicated to developing a reversible BiFC system. In conclusion, the development of TagBiFC for the visualization and tracking of protein complexes in living cells at the nanometer and millisecond scale will facilitate significant insights into protein interaction studies, expanding our toolkits for the investigation of PPIs at their native state.

## Methods

### Plasmid construction

All primers used in this work were ordered from Ruibo Biotech (Beijing). The (GGS)_X6_ liner sequence was directly synthesized and ligated into plasmid pcDNA3.1(+) to get pcDNA3.1(+)-(GGS)_X6_. The leucine zipper domains from β-Fos were amplified from human cDNA (reversed transcript from MAD-MB-231 cells) and ligated into plasmid pcDNA3.1(+)-(GGS)_X6_ via enzyme Hind III and BamH I (NEB). The β-Fos (ΔZIP) sequence was generated by PCR and ligated into pcDNA3.1(+)-(GGS)_X6_ to get pcDNA3.1(+)-c-Fos (ΔZIP)-(GGS)_X6_. The leucine zipper domains from β-Jun were constructed in the similar way but with enzyme EcoR I and XbaI (NEB). The HaloTag plasmid was purchased from Promega Corporation (#G9651) and used as a template to amplify different split HaloTagN and HaloTagC pairs. All the HaloTagN fragments were inserted into pcDNA3.1(+)-(GGS)_X6_-β-Jun with enzyme Hind III and BamH I (NEB), while all the HaloTagC fragments were inserted into pcDNA3.1(+)-β-Fos-(GGS)_X6_ with enzyme EcoR I and XbaI (NEB). The split mMaple3 plasmids (from Professor Xiaowei Zhuang’s Laboratory, mMaple3 N173 and mMaple3 C173) were conducted by replacing HaloTagN and HaloTagC in the pcDNA3.1(+)-β-Fos-(GGS)_X6_-HaloTagC and pcDNA3.1(+)-HaloTagN-(GGS)_X6_-β-Jun via the corresponding enzymes. The FKBP and FRB fragments were amplified from custom plasmid and replaced the β-Fos and β-Jun sequences in pcDNA3.1(+)-HaloTagN-(GGS)_X6_-β-Jun and pcDNA3.1(+)-β-Jun-(GGS)_X6_-HaloTagC. The full length of c-Fos and c-Jun were amplified from human cDNA (reversed transcript from MAD-MB-231 cells) and constructed in a similar manner. Different mutants of c-Fos and c-Jun were constructed by PCR and replaced the full-length cDNA in the corresponding plasmids. More detailed information about the plasmids is included in Supplementary Information as Supplementary Table 1.

All the digestion processes were conducted at 37 °C for 1 h, while all the ligation processes were conducted at 16 °C for 30 min. *E. coli* (Trans5α, bought from TransGene, China) was used for all the transformations.

### Cell culture, transfection, and labeling

Human breast cancer cell line MDA-MB-231 were maintained in Dulbecco’s modified Eagle medium with high glucose (Lifetech), 10% fetal bovine serum (Lifetech), and 1× penicillin/streptomycin (Lifetech). Cells were maintained at 37 °C and 5% CO_2_ in a humidified incubator. One day before transfection, cells were passaged into 35-mm petri dish with about 50% density. All plasmids were transiently transfected with Lipofectamine 2000 (Lifetech) in accordance with the manufacturer’s protocol.

For both live and fixed single-molecule experiments, whole-cell imaging, and FACS, cells were grown overnight on 35-mm petri-dish. After overnight growth, cells were labeled with the relevant Halo-JF549 dye at a final concentration of 0.1 nM for 15 min and then washed three times with 1× phosphate-buffered saline (PBS). After the final wash, PBS were replenished with fresh phenol red-free medium (Lifetech) for imaging or in PBS for FACS.

### Flow cytometry

Control or transfected MDA-MB-231 cells were incubated in medium containing 0.1 nM JF549 for 15 min and then washed with 1× PBS for 3 times and collected in 0.25% trypsin for 2 min, spun down for 3 min at 500 × *g*, and resuspended in 1× PBS. Flow cytometry was performed on a BD LSR Fortessa. Cells were gated for viability and single cells. All the parameters remained constant within parallel experimental runs for comparation. All flow cytometric data were analyzed with FlowJo.

### Western blotting

Cells were lysed in RIPA Lysis Buffer (Beyotime, P0013B) with protease inhibitor PMSF (Beyotime, ST505). Lysate was run on a 4–20% Tris-acetate gel (Yeasen, Precast Protein Improve Gels, 36231ES10) at 70 V for 1 h, followed by 100 V until dye front reached the end of the gel. Protein was then wet transferred to a 0.45-mm polyvinylidene fluoride membrane (Millipore, IPVH00010) in ice-cold transfer buffer (25 mM Tris, 200 mM glycine, 10% methanol) at 200 mA for 2 h at room temperature. After transfer, the membrane was blocked and shook with 5% non-fat milk in TBST for 1 h at room temperature. Membrane was then incubated with the corresponding antibodies (c-Fos (9F6) rabbit monoclonal antibody (mAb) CST#2250 (dilution 1:1000), c-Jun (60A8) rabbit mAb CST#9165 (dilution 1:1000), Flag (D6W5B) Rabbit mAb CST#14793 (dilution 1:500), GAPDH mouse monoclonal (Proteintech-60004-1-Ig, dilution 1:50,000)) diluted in 5% non-fat milk in TBST and incubated for 2 h at room temperature. Then the membrane was washed three times with TBST for 5 min at room temperature and shook for each wash. Membrane was incubated with 1:5000 secondary antibodies (anti-mouse IgG, horseradish peroxidase (HRP)-linked antibody #7076, dilution 1:3000 and anti-rabbit IgG, HRP-linked antibody #7074, dilution 1:3000) for 1 h at RT and washed three times in TBST for 5 min. Membranes were covered with ECL substrate (Thermo Scientific, 34080) and imaged using a CCD camera.

### RNA FISH

We used the Cy5-labeled secondary probe coupled with unlabeled primary probes to perform CFP single-molecule FISH. We designed 32 primary probes (Supplementary Table 2) covering the coding region of CFP. The hybridization of the primary probe set with the secondary probe was performed in NEB3.1 buffer in a PCR machine using the conditions of 85 °C 3 min, 65 °C 3 min, and 25 °C 5 min. The cells were permeabilized in 70% ethanol overnight at 4 °C, with a parafilm sheet wrapped around the dishes. Then the cells were rinsed once with PBS and incubated in 15% formamide freshly prepared in 1× saline-sodium citrate (SSC, Ambion, AM9763) buffer for 15 min at room temperature. Two hundred-microliter mixer was prepared containing 10 μL 20× SSC, 4 μL 20 μg/μL *E. coli* tRNA, 30 μL 100% formamide, 4 μL annealed probes, 2 μL 20 mg/mL RNAse-free bovine serum albumin, 2 μL 200 mM VRC, 53 μL 40% dextran sulfate, and 96 μL DEPC-treated water on ice. Then the PBS was removed from the dishes, and the mixer was added to the cells. The petri dish was wrapped using a parafilm and incubated at 37 °C overnight in a humidified incubator. On the second day, the hybridization mixer was removed, and the cells were washed using freshly prepared 15% formamide in 1× SSC at 37 °C for 30 min. The cells were rinsed twice in PBS.

### Image acquisition

Briefly, all images were taken on a custom Olympus IX83 inverted microscope equipped with a ×100 UPlanSApo, N.A. = 1.49, oil-immersion phase objective, and Andor iXon Ultra EMCCD. The microscope stage incubation chamber was maintained at 37 °C and 5% CO_2_. A 405-nm laser was used to activate mMaple3; a 561-nm laser was used to excite mMaple3 and JF549. The laser power was modulated by an acousto-optic tunable filter. In all experiments, we used highly inclined thin illumination (HILO) and carefully optimized the angle of the inclined light to reduce background from cell auto-fluorescence in the live cell tracking experiment. For whole-cell imaging, we transfected the cells with corresponding plasmids for about 24 h and then incubated the cells in medium containing 0.1 nM JF549 for 15 min. After washing with PBS for 3 times, the cells were fixed with 4% paraformaldehyde for 15 min and washing with PBS and then stained with 4,6-diamidino-2-phenylindole for 5 min. Then the cells were imaged under wide-field microscopy using ~1 mW 561 nm and 405 nm laser power and 500 ms exposure time. For single-molecule imaging, to get sparse labeling, cells were pre-bleached with high 561 nm laser power (~35 mW) to bleach most of the labeled molecules for 5–10 s and the left molecules were resolved without overlap. Then single-molecule movies were taken using 10 mW 561 nm laser power and 10 ms exposure time.

### Diffusion components and chromatin-binding fraction calculation

All the single-molecule imaging data were analyzed by ImageJ plugin Trackmate^54^. Then the molecule coordinates were analyzed by custom R scripts in RStudio. Analysis of MSD was carried out using custom MATLAB scripts, which has been described previously^55^. To quantify the localization precision of single molecules of split HaloTag and mMaple3, we fitted each molecule with two-dimensional Gaussian function. The localization error is calculated as followed:

$$\left( {x,y} \right) = B + \frac{A}{{2\pi\sigma _x\sigma _y}} \times e^{ - \left( {\frac{{\left( {x - x_0} \right)^2}}{{2\sigma _x^2}}} \right) - \left( {\frac{{\left( {y - y_0} \right)^2}}{{2\sigma _{\mathrm{y}}^2}}} \right)}$$

$$s = \frac{{\sigma _x + \sigma _y}}{2}$$

where *B* is the background level, *A* is the total volume of the Gaussian, *x*_0_ is the *x* center of the Gaussian, *y*_0_ is the *y* center of the Gaussian, ${\sigma}_x$ is the *x* standard deviation, ${\sigma}_y$ is the *y* standard deviation, and *s* is the localization error.

Then the maximum likelihood diffusion coefficient (*D*) per track was calculated using the following formula:

$$D = \frac{{\Delta x^2 + \Delta y^2}}{{4 \ast \Delta t}}$$

where $\Delta x$ and $\Delta y$ is averaged step size between successive frames and $\Delta t$ is the time interval between successive frames. Each trajectory only gives one averaged *D* value in the analysis method, thus ensuring that the stable chromatin-bound fraction of the TFs is not overestimated.

We used the Gaussian distribution function of two components to fit the distribution of diffusion coefficient (Log*D*):

$$y = \frac{{A1}}{{w1 \times \sqrt {\frac{\pi }{2}} }}e^{\frac{{ - 2\left( {x - x1} \right)}}{{w1^2}}} + \frac{{A2}}{{w2 \times \sqrt {\frac{\pi }{2}} }}e^{\frac{{ - 2\left( {x - x2} \right)}}{{w2^2}}}$$

where *y* is the frequency distribution, *A* is the area of the Gaussian peak, *w* is the half height and full width, *x*1 and *x*2 are the center of the Gaussian peak.

### Residence time calculation

To calculate residence time and survival probability of molecules on chromatin, we performed time-lapse illumination with a 10-ms camera integration time interspersed with a dark period of 490 ms. We then set a threshold to select the chromatin-bound TF molecules based on the maximum likelihood diffusion coefficient of their trajectories. The Log*D* threshold (*μ* + 2*σ*, 95%) was calculated using parameters *μ* (mean) and *σ* (standard deviation), which are derived from the Gaussian distribution of chromatin-binding fraction in the previous step. In our example, Log*D* < 0.50 μm^2^/s was used to choose chromatin-bound molecules.

The apparent residence time was directly calculated based on the duration of individual tracks.

$$t_{{\rm{app}}} = N * t_{{\rm{interval}}}$$

where $t_{{\rm{app}}}$ is the apparent residence time, *N* is the length of each trajectory, and $t_{{\rm{interval}}}$ is the interval time between successive frames (500 ms).

Photobleaching correction was implemented as previously described^30^. Briefly, the cells with same HaloTag-labeled molecules were imaged under the same excitation conditions without pre-bleaching. The photobleaching decay can be directly estimated by plotting the total fluorescence of the whole cells as a function of time. The intensity decay can be fitted to a bi-exponential decay:

$$F = y_o + f_{b1} \ast e^{ - \left( {\frac{t}{{t_{b1}}}} \right)} + f_{b2} \ast e^{ - (\frac{t}{{t_{b2}}})}$$

where $y_o$ is offset, $f_{b1}$ and $f_{b2}$ are amplitudes, and $t_{b1}$ and $t_{b2}$ are photobleaching time constants.

To avoid the influence of photobleaching on the calculation of residence time, we normalized the cumulative frequency distributions (CDFs) of dwell times by dividing them with the photobleaching decay function $\frac{F}{{F(0)}}$.

$${\mathrm{CDF}}_{{\rm{corr}}} = {\mathrm{CDF}}(t_{{\rm{app}}}) \ast \frac{{F(0)}}{F}$$

The survival probability is described as complement cumulative density function 1 − CDF, which is then fit with a two-component exponential decay:

$$1 - {\rm{CDF}}_{{\rm{corr}}} = y_o + f_1^\ast e^{ - \left( {\frac{t}{{t_1}}} \right)} + f_2^\ast e^{ - (\frac{t}{{t_2}})}$$

where $y_o$ is offset, $f_1$ and $f_2$ are amplitudes, and $t_1$ and $t_2$ are specific and non-specific residence times.

The long-lived (specific) bound population:

$$F_{\rm{s}} = \frac{{f_2}}{{f_2 + f_1}}$$

The short-lived (non-specific) bound population:

$$F_{{\rm{ns}}} = \frac{{f_1}}{{f_2 + f_1}}$$

### Statistics and reproducibility

All the fluorescence images were repeated at least once at a different time or by a different researcher. The error bars shown in the text (Figs. 2 and 4) were derived from three independent experiments and fittings. The *p* value statistics of Fig. 5j is 2.193e–06, paired *t* test, *n* = 16 for the +Dox group and *n* = 23 for the −Dox group.

### Reporting summary

Further information on research design is available in the Nature Research Reporting Summary linked to this article.

## Supplementary information

Supplementary Information

Description of Supplementary Files

Supplementary Data 1

Supplementary Data 2

Supplementary Data 3

Supplementary Data 4

Supplementary Data 5

Reporting Summary

## Data Availability

The data that support the findings of this study are available from the corresponding author upon reasonable request. The Source data underlying Fig. 1c, g are provided as Supplementary Data 1. The Source data underlying Fig. 2b–d are provided as Supplementary Data 2. The Source data underlying Fig. 3a–j are provided as Supplementary Data 3. The Source data underlying Fig. 4c–k are provided as Supplementary Data 4. The Source data underlying Fig. 5d, j are provided as Supplementary Data 5.
