# Supplementary material for: TagBiFC technique allows long-term single-molecule tracking of protein-protein interactions in living cells
Source: Commun Biol. 2021 Mar 19;4:378. doi: 10.1038/s42003-021-01896-7 (PMC7979928; doi:10.1038/s42003-021-01896-7)
Supplement: Supplementary file 1 — Supplementary Information [file 42003_2021_1896_MOESM1_ESM.pdf]

Supplementary Information

TagBiFC technique allows long-term single-molecule tracking of protein-protein interactions in living cells

Shipeng Shao[1,2], Hongchen Zhang[1], Yong Zeng[2], Yongliang Li[1,3], Chaoying Sun[1], Yujie Sun[1,]*

[1] State Key Laboratory of Membrane Biology, Biomedical Pioneering Innovation Center (BIOPIC), School of Life Sciences, Peking University, Beijing 100871, China.

[2] Beijing Institute of Heart Lung and Blood Vessel Disease, Beijing Anzhen Hospital, Capital Medical University, Beijing, 100029, China

[3] The second Dental Center, Peking University School and Hospital of Stomatology, Beijing 100871, China.

**E-mail: sun_yujie@pku.edu.cn*

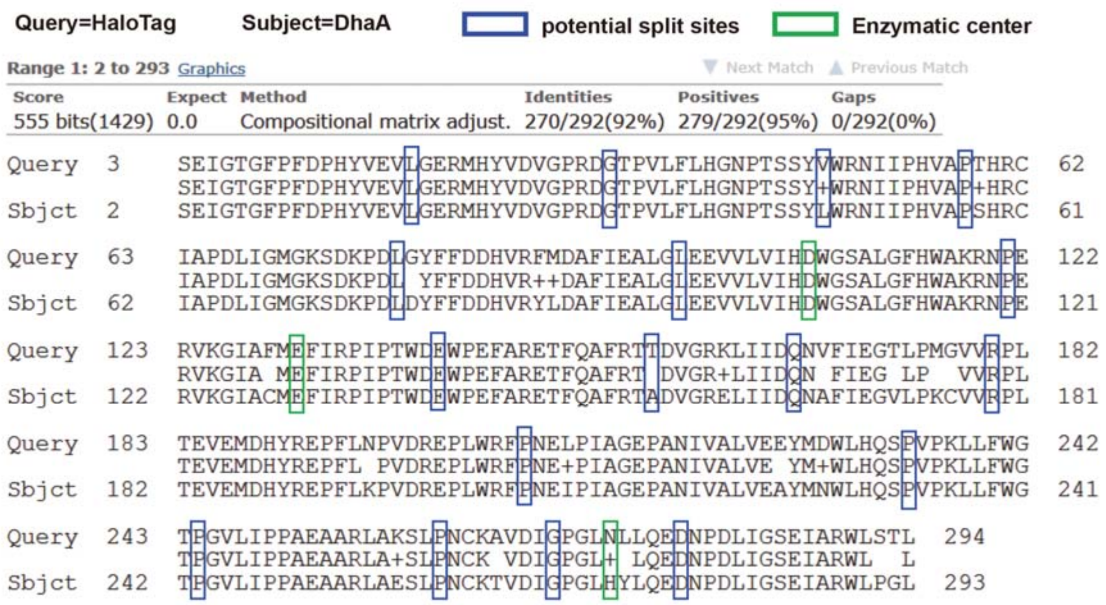

Supplementary Figure 1. The sequence alignment of HaloTag and DhaA.

The Query sequence is HaloTag and the subject sequence is DhaA. The enzymatic center consisting of three amino acids were boxed in green. The potential split sites of HaloTag were boxed in blue. Each of the split sites were in flexible loops between two secondary structures units according to the three-dimensional crystal structure.

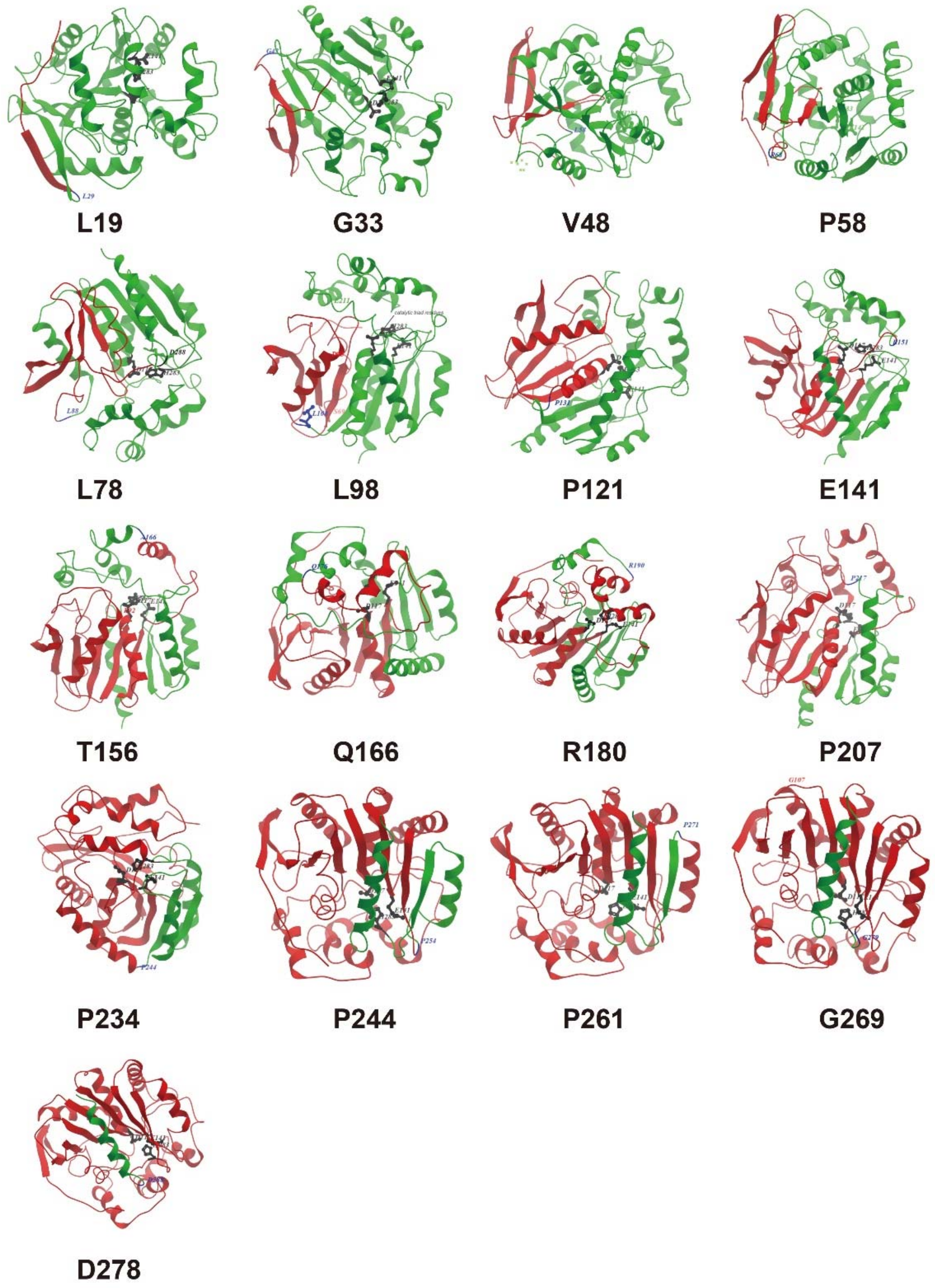

Supplementary Figure 2. Three-dimensional structure diagram of all the 17 split sites of HaloTag.

The displayed structures are haloalkane dehalogenase DhaA (1BN6). The labeled split site in the images are number form haloalkane dehalogenase DhaA. The words under each figure are number form HaloTag. Three amino acids that make up the enzymatic center are labeled in gray. The N terminal of the split protein is labeled in green while the C terminal of the split protein is labeled in red.

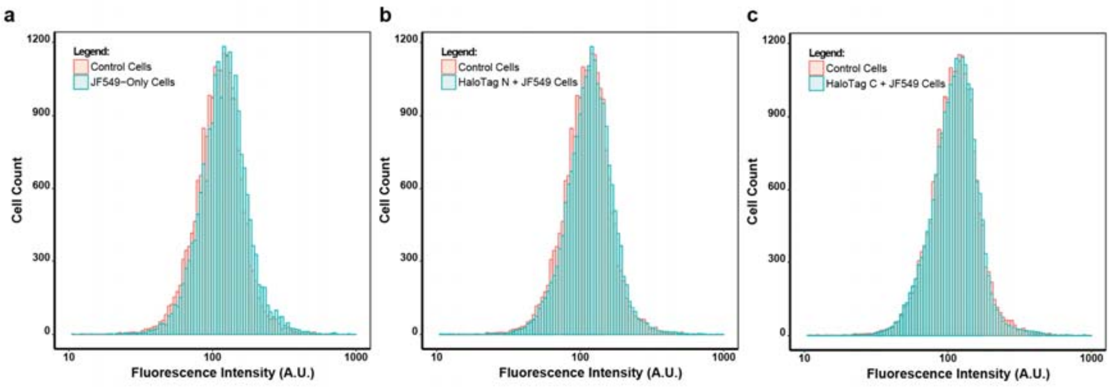

Supplementary Figure 3. Neither HaloTag N58 nor HaloTag C58 has residual enzymatic activity.

Cells without HaloTag **(a)**, or with HaloTag N58 **(b)**, or HaloTag C58 **(c)** expression were kept in medium containing 0.1 nM JF549 for 15 minutes and then washed with PBS for 3 times. Control cells were treated with equal amount DMSO. Whole-cell fluorescence intensity was analyzed using FACS. The nearly identical fluorescence intensity of these two groups demonstrated that the JF549 dye was not retained in cells without HaloTag. The super membrane permeability of JF549 dye is the guarantee for the successful single molecule imaging in the following experiments since single molecule detection is more sensitive to background fluorescence.

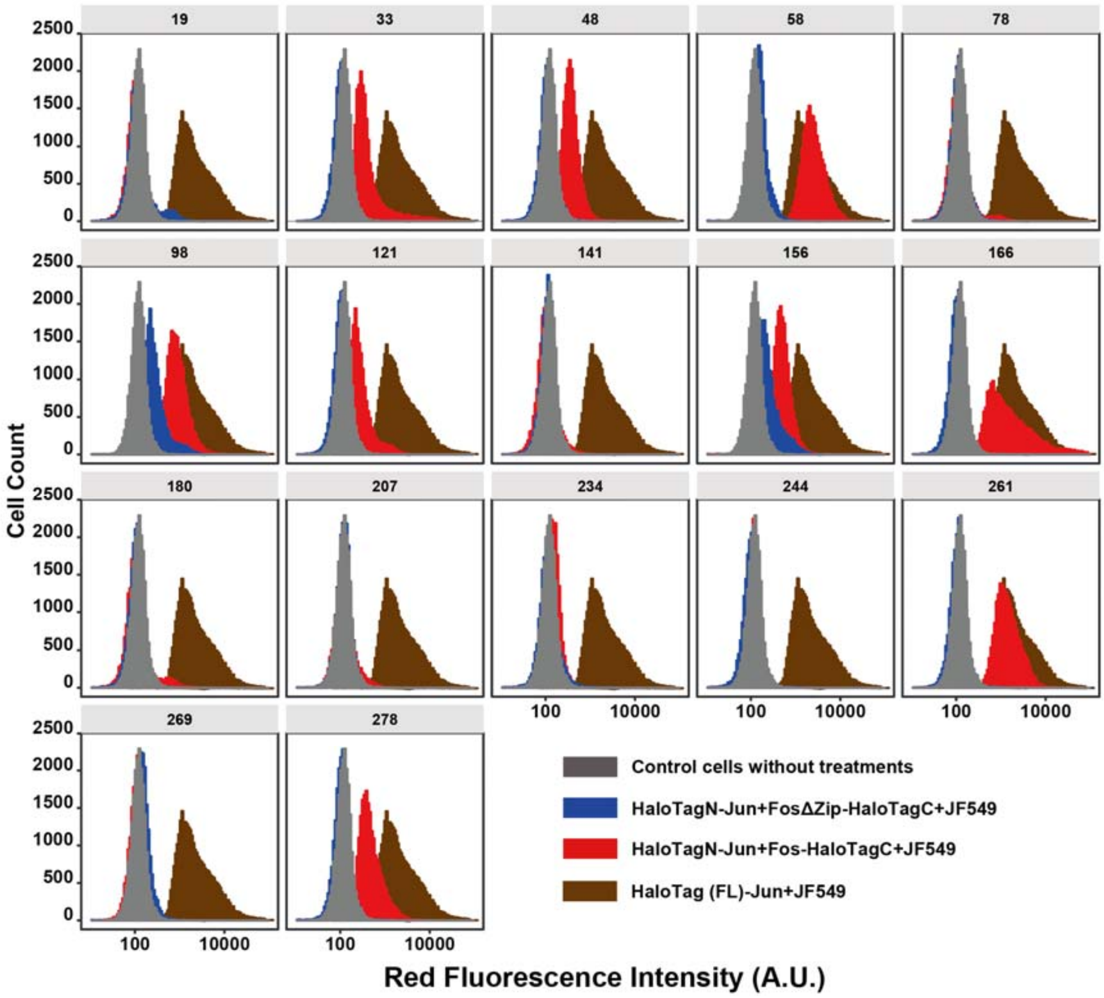

Supplementary Figure 4. Fluorescent intensity retained within cells of different split sites.

For all the potential split sites, interaction protein pair Fos and Jun, not interaction pair Fos (ΔZIP) and Jun were fused with split HaloTag, respectively. Cells were kept in medium containing 0.1 nM JF549 for 15 minutes and then washed with PBS for 3 times. All the data were collected using FACS. Blank cells and cells transfection with full-length (FL) HaloTag were used as controls. Fluorescent intensity of more than 10000 cells were collected in one experiment.

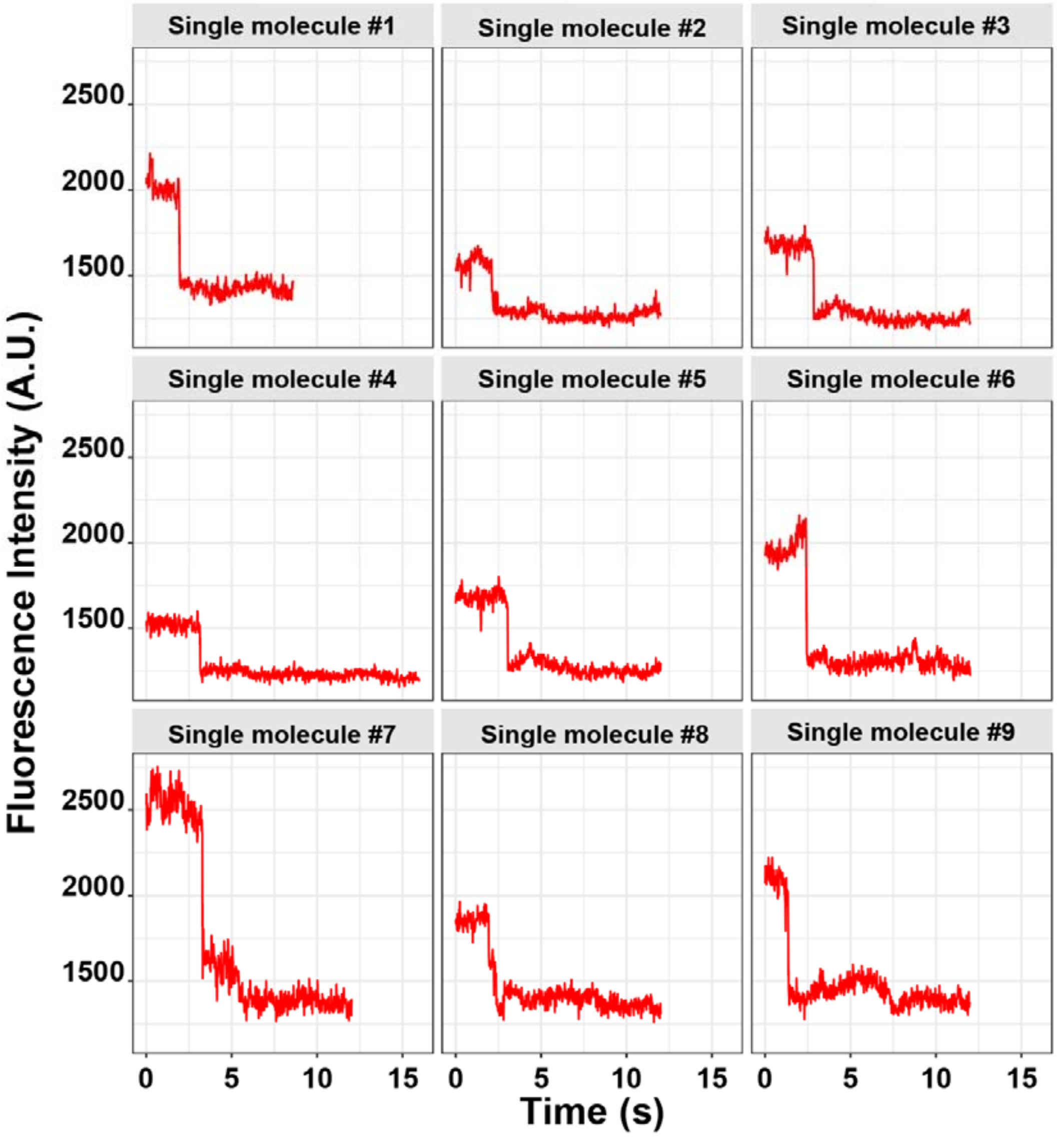

Supplementary Figure 5. The reconstituted split HaloTag remains single molecule property.

Fluorescent intensity of nine random chosen molecules. All nine molecules displayed only one plunge during the image acquisition. One single step photo-bleaching of the intensity also indicated that they were indeed single molecules.

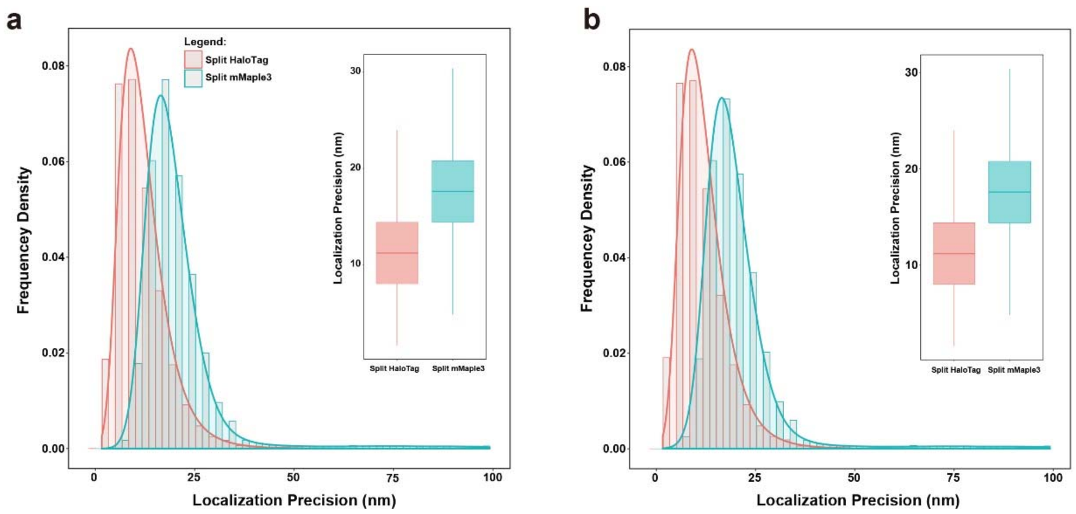

Supplementary Figure 6. Comparison of single molecule localization precision of split HaloTag and split mMaple3.

(a). Histogram of x localization precision of split HaloTag and split mMaple3. Inset is the boxplot of the same data.
(b). Histogram of y localization precision of split HaloTag and split mMaple3. Inset is the boxplot of the same data.

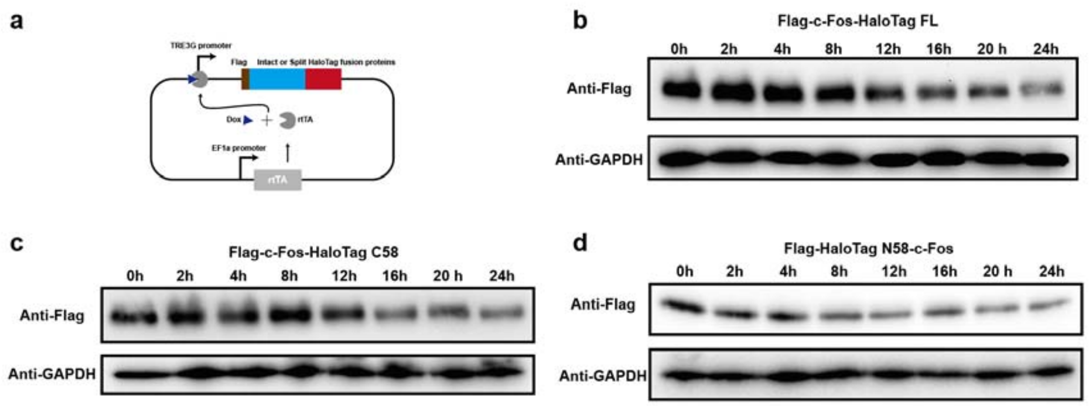

Supplementary Figure 7. Comparison of stability of intact HaloTag and split HaloTag.

(a). Schematic of expression plasmid of intact HaloTag and split HaloTag. Two expression cascades were cloned into one plasmid to ensure the co-appearance in the same cells. The rtTA was driven by EF1a promoter while the intact HaloTag or split HaloTag fusion proteins was driven by tetracycline responsible element (TRE) promoter. Flag tag (DYKDDDDK) was also fused to the N-terminal of all the proteins for the convenience of detection using western blot.

(b-d). Western blot showing the decay process of intact or split HaloTag fusion proteins. HeLa cells were transfected with the corresponding constructs for 24 h. After the induction using 10 μg/ml Doxycycline Hyclate for 6 h, Doxycycline was withdrawn to shut down the expression of intact HaloTag and split HaloTag. Then we tracked the level of intact HaloTag and split HaloTag using western blot at different time point.

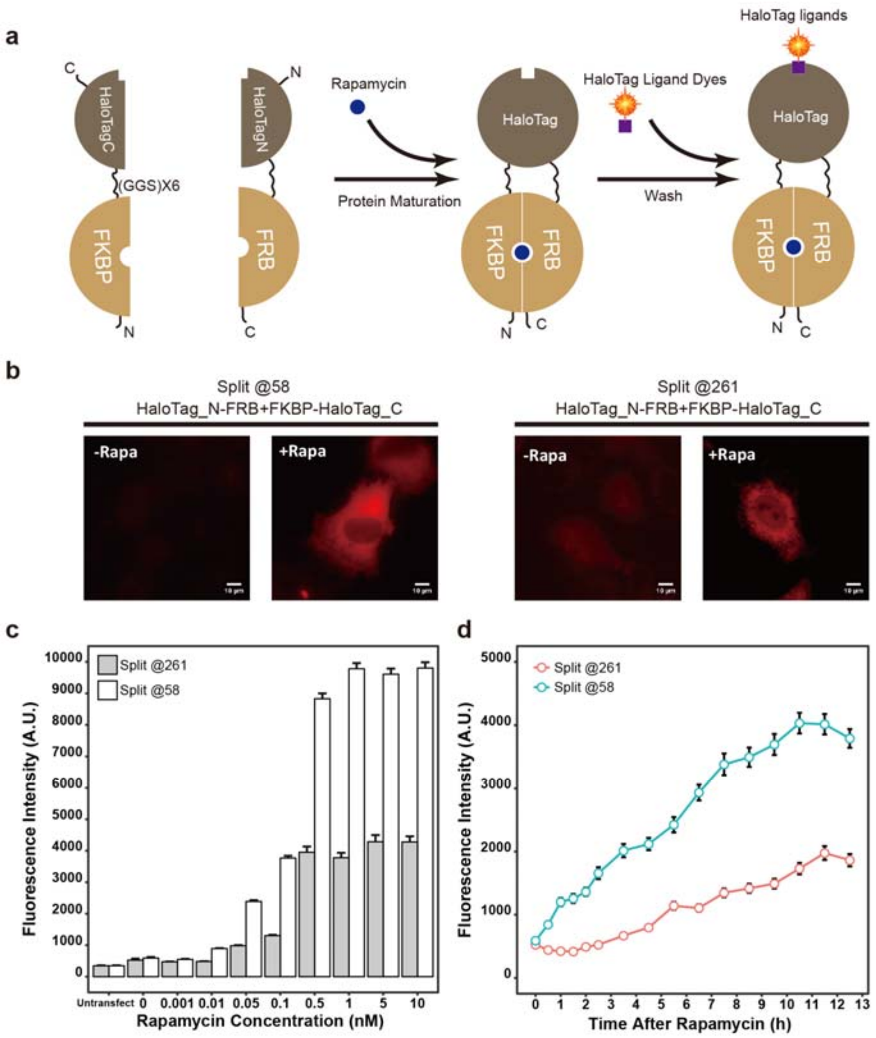

Supplementary Figure 8. Measure the maturation process of split HaloTag.

(a). Schematic diagram for the measurement of split HaloTag maturation. FKBP and FRB were fused with split HaloTag respectively. When Rapamycin is added to the cells, the split HaloTag will form intact HaloTag and bind its ligand.

(b). Fluorescence images of FKBP and FRB fused with split HaloTag at 58 or 261 split sites with or without Rapamycin.

(c). The BiFC efficiency of split HaloTag at 58 or 261 split sites under different Rapamycin concentrations. The data were generated using FACS and more than 10000 cells were collected in one experiment. Error bars represent standard deviation (SD) of three independent experiments.

(d). The BiFC efficiency of split HaloTag at 58 or 261 split sites as a function of induction time under fixed Rapamycin concentration (0.5 nM). The data were generated using FACS and more than 10000 cells were collected in one experiment. Error bars represent standard deviation (SD) of three independent experiments.

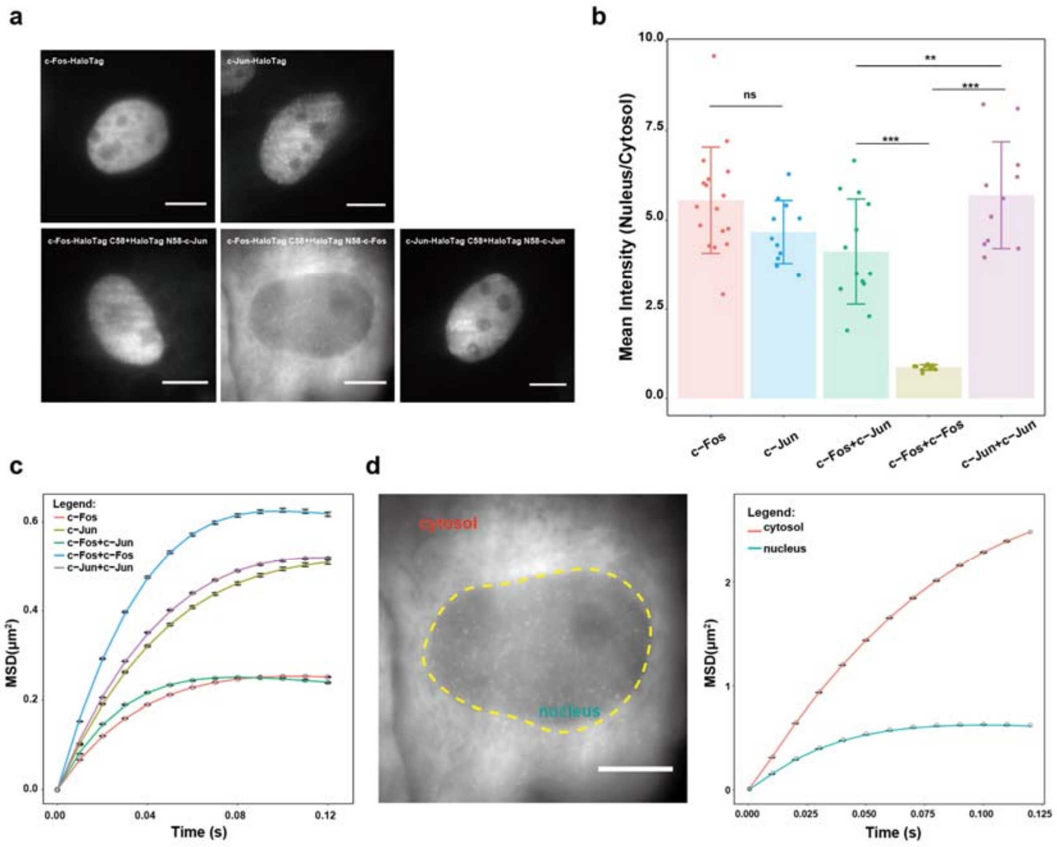

Supplementary Figure 9. Both c-Fos and c-Jun can form dimer in living cells.

(a). Fluorescence images of c-Fos and c-Jun homodimers and heterodimers. c-Fos or c-Jun fused with intact HaloTag were shown in the upper panel while c-Fos homodimer, c-Jun homodimer, and c-Fos+c-Jun heterodimer labeled using split HaloTag were shown in the lower panel. All scale bars are 5 μm.

(b). Nuclear enrichment index (Nucleus/Cytosol fluorescence intensity) of different c-Fos and c-Jun dimers in living cells. **Statistically significant difference ($P < 0.01$, paired t test). ***Statistically significant difference ($P < 0.001$, paired t test). ns, non-significant.

(c). Single molecule mean square displacement of different c-Fos and c-Jun dimers in living cells. Error bars represent standard deviation (SD). The data were fit by power law function $MSD = \mathrm{A} * t^{a}$.

(d). Left panel: Fluorescence image of c-Fos and c-Jun heterodimer labeled by split HaloTag. The scale bar is 5 μm. Right Panel: Single molecule mean square displacement of c-Fos and c-Jun heterodimer in the nucleus and in the cytosol. Error bars represent standard deviation (SD). The data were fit by power law function $MSD = \mathrm{A} * t^{a}$.

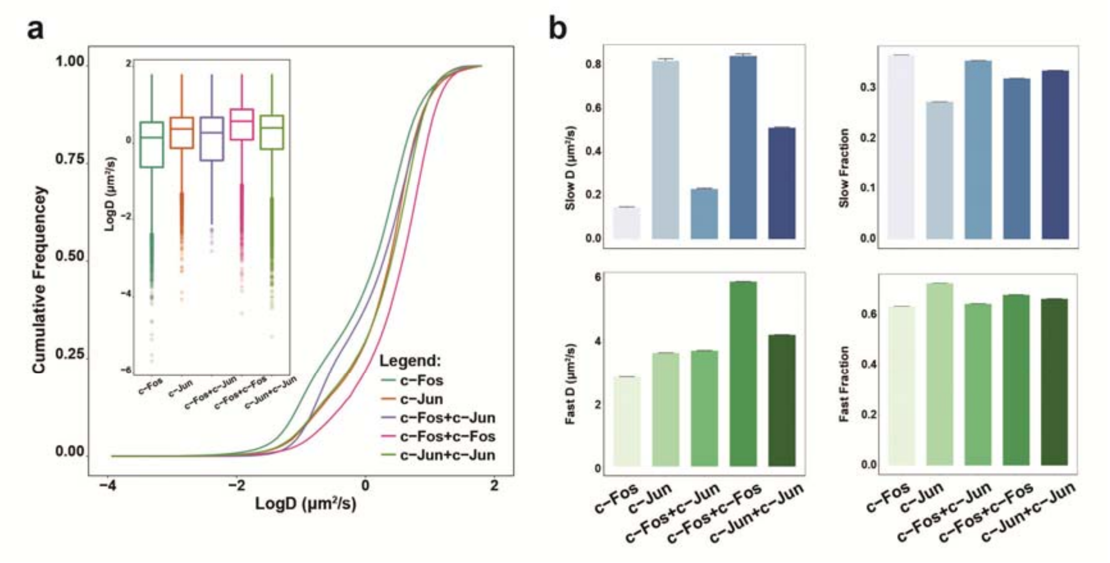

Supplementary Figure 10. Diffusion behaviors of different c-Fos and c-Jun combinations.

(a). Cumulative distribution of diffusion constant (D) of combinations of c-Fos and c-Jun dimers. Inset showed the boxplot of the same data.

(b). Fast and slow diffusion constant (D) and fraction of combinations of c-Fos and c-Jun dimers. The data were generated by fitting the distribution of diffusion constant with two components Gaussian function. Error bars represent 95% confidence interval of the fitting.

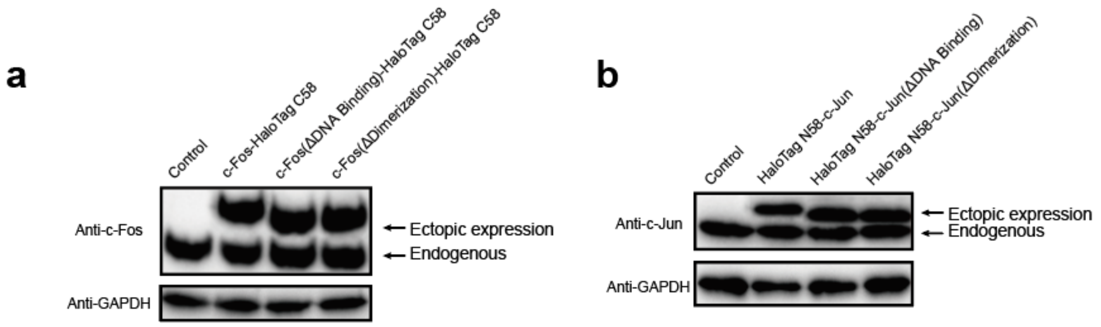

Supplementary Figure 11. The expression level of ectopic TagBiFC labeled proteins were similar to the endogenous ones.

Western blot showing that TagBiFC fusion c-Fos (a) and c-Jun (b) have similar expression level to endogenous c-Fos and c-Jun. HeLa cells were transfected with the corresponding constructs for 24 h before western blot was performed. The untransfected cells were used as control.

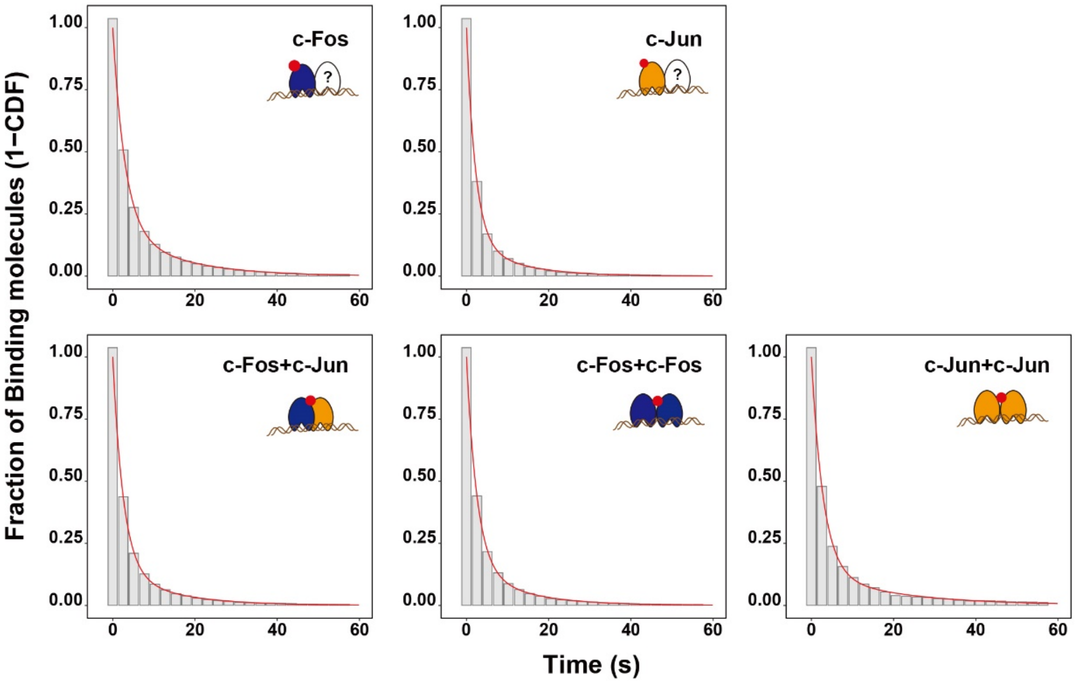

Supplementary Figure 12. Residence time of different c-Fos and c-Jun combinations.

c-Fos and c-Jun were fused with intact HaloTag while c-Fos homodimer, c-Jun homodimer, and c-Fos+c-Jun heterodimer were labeled using split HaloTag. The data were fit by two components exponential decay function.

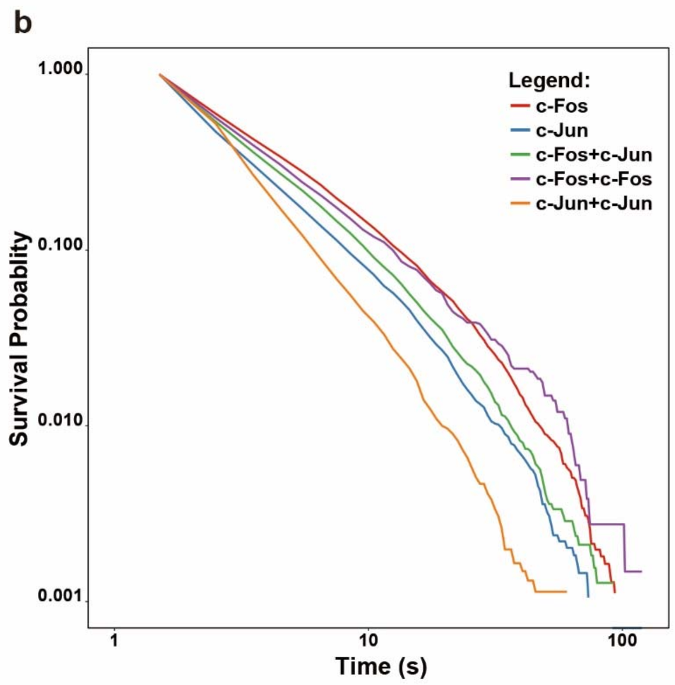

Supplementary Figure 13. Survival probability of different c-Fos and c-Jun combinations.

c-Fos or c-Jun were fused with intact HaloTag while c-Fos homodimer, c-Jun homodimer, and c-Fos+c-Jun heterodimer were labeled using split HaloTag.

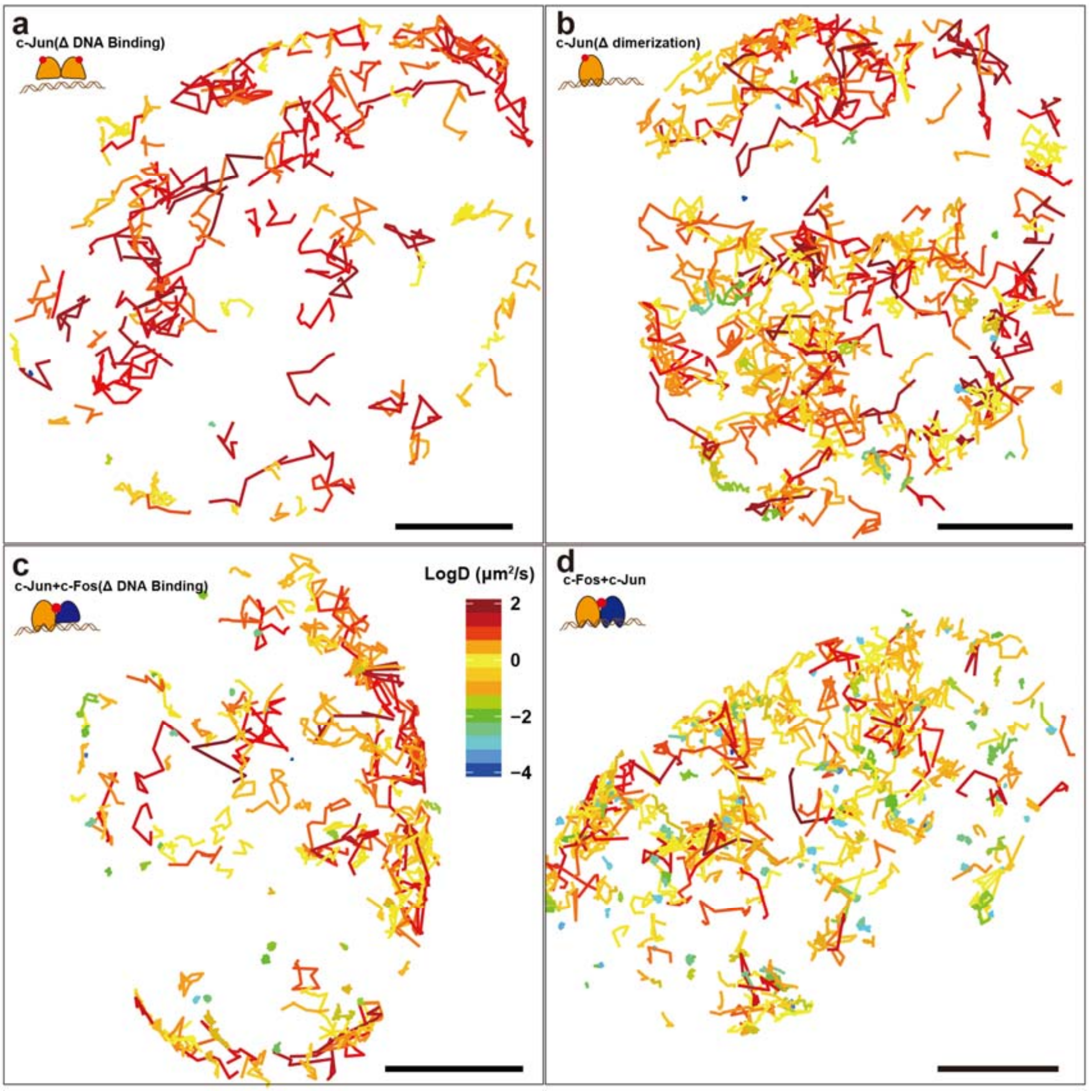

Supplementary Figure 14. Single molecule trajectories of different c-Jun mutants.

(a). Single molecule trajectories of c-Jun mutant incapable of binding to DNA but capable of forming dimer.

(b). Single molecule trajectories of c-Jun mutant incapable of forming dimer but capable of binding to DNA.

(c). c-Fos incapable of binding to DNA was forced to form dimer with c-Jun using split HaloTag.

(d). Wild type c-Fos was forced to form dimer with c-Jun using split HaloTag. The color of the trajectories represented different diffusion constants.

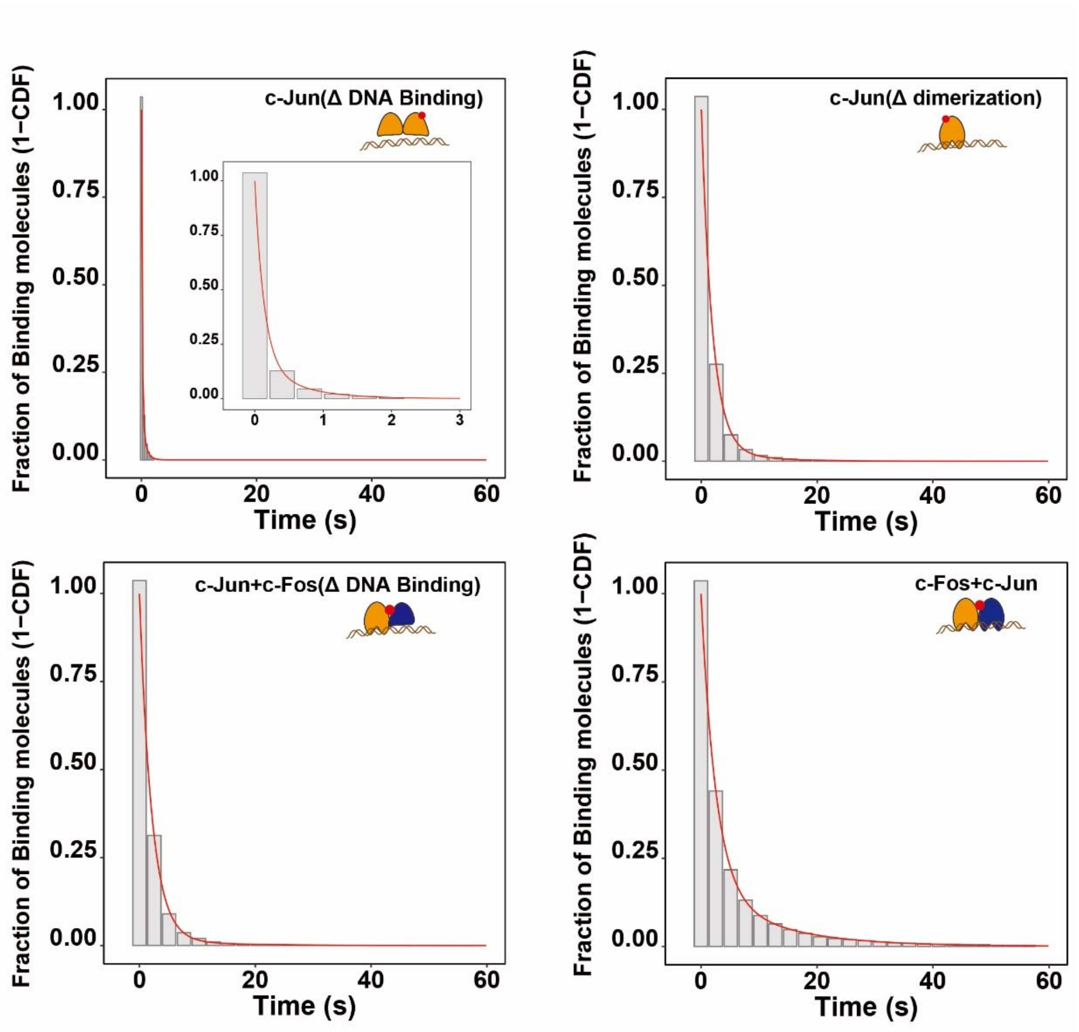

Supplementary Figure 15. Residence time of different c-Jun mutants.

c-Jun mutant incapable of binding to DNA but capable of forming dimer was fused with intact HaloTag. c-Jun mutant incapable of forming dimer but capable of binding to DNA was fused with intact HaloTag. c-Fos incapable of binding to DNA was forced to form dimer with c-Jun using split HaloTag. Wild type c-Fos was forced to form dimer with c-Jun using split HaloTag.

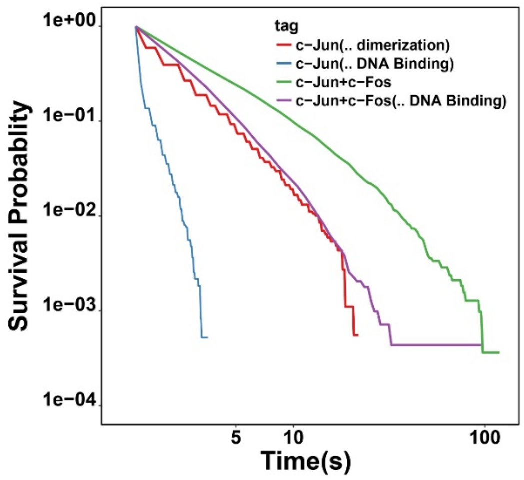

Supplementary Figure 16. Survival probability of different c-Jun mutants.

c-Jun mutant incapable of binding to DNA but capable of forming dimer was fused with intact HaloTag. c-Jun mutant incapable of forming dimer but capable of binding to DNA was fused with intact HaloTag. c-Fos incapable of binding to DNA was forced to form dimer with c-Jun using split HaloTag. Wild type c-Fos was forced to form dimer with c-Jun using split HaloTag. The color of the trajectories represented different diffusion constant.

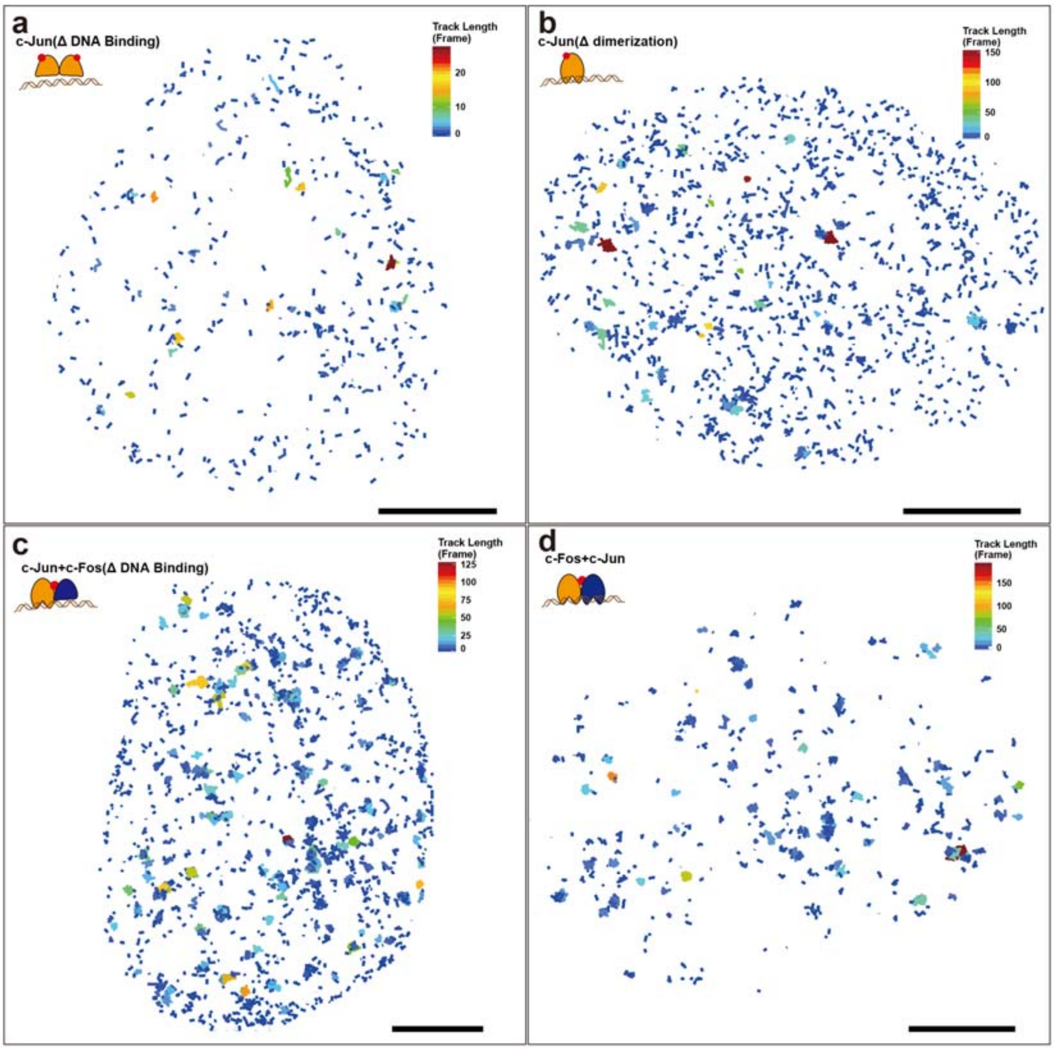

Supplementary Figure 17. Single molecule trajectories of different c-Jun mutants that stable bind to chromatin.

(a). Single molecule trajectories of c-Jun mutant incapable of binding to DNA but capable of forming dimer. The images were taken using long exposure time to filter out the free diffused molecules.

(b). Single molecule trajectories of c-Jun mutant incapable of forming dimer but capable of binding to DNA.

(c). c-Fos incapable of binding to DNA was forced to form dimer with c-Jun using split HaloTag.

(d). Wild type c-Fos was forced to form dimer with c-Jun using split HaloTag. The color of the trajectories represented different trajectory length.

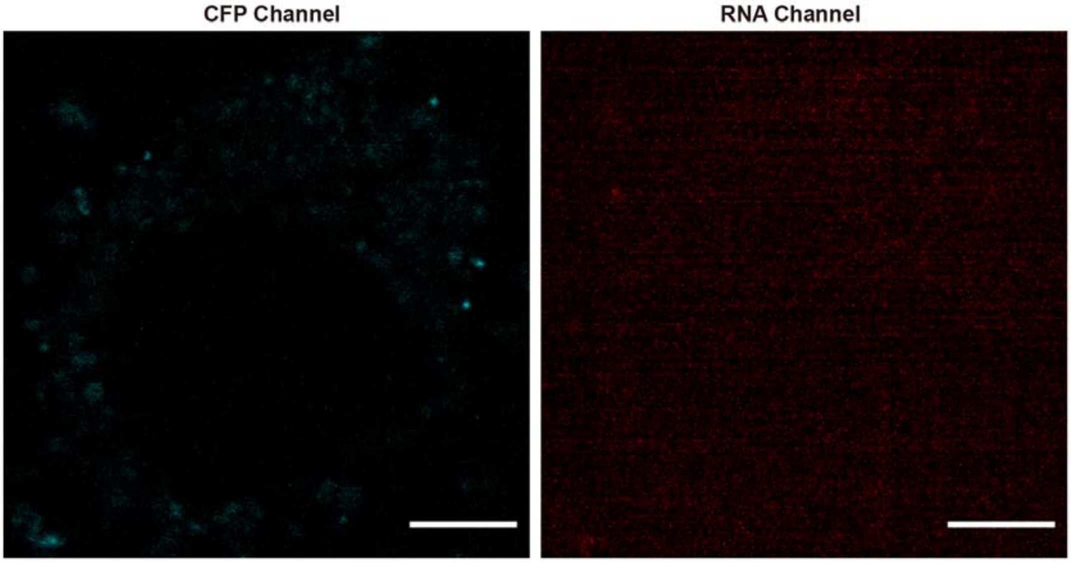

Supplementary Figure 18. Specificity of mRNA labeling using TagBiFC.

In the control cells without ECFP-1×MS2-PP7 transfection, no fluorescent puncta could be monitored.

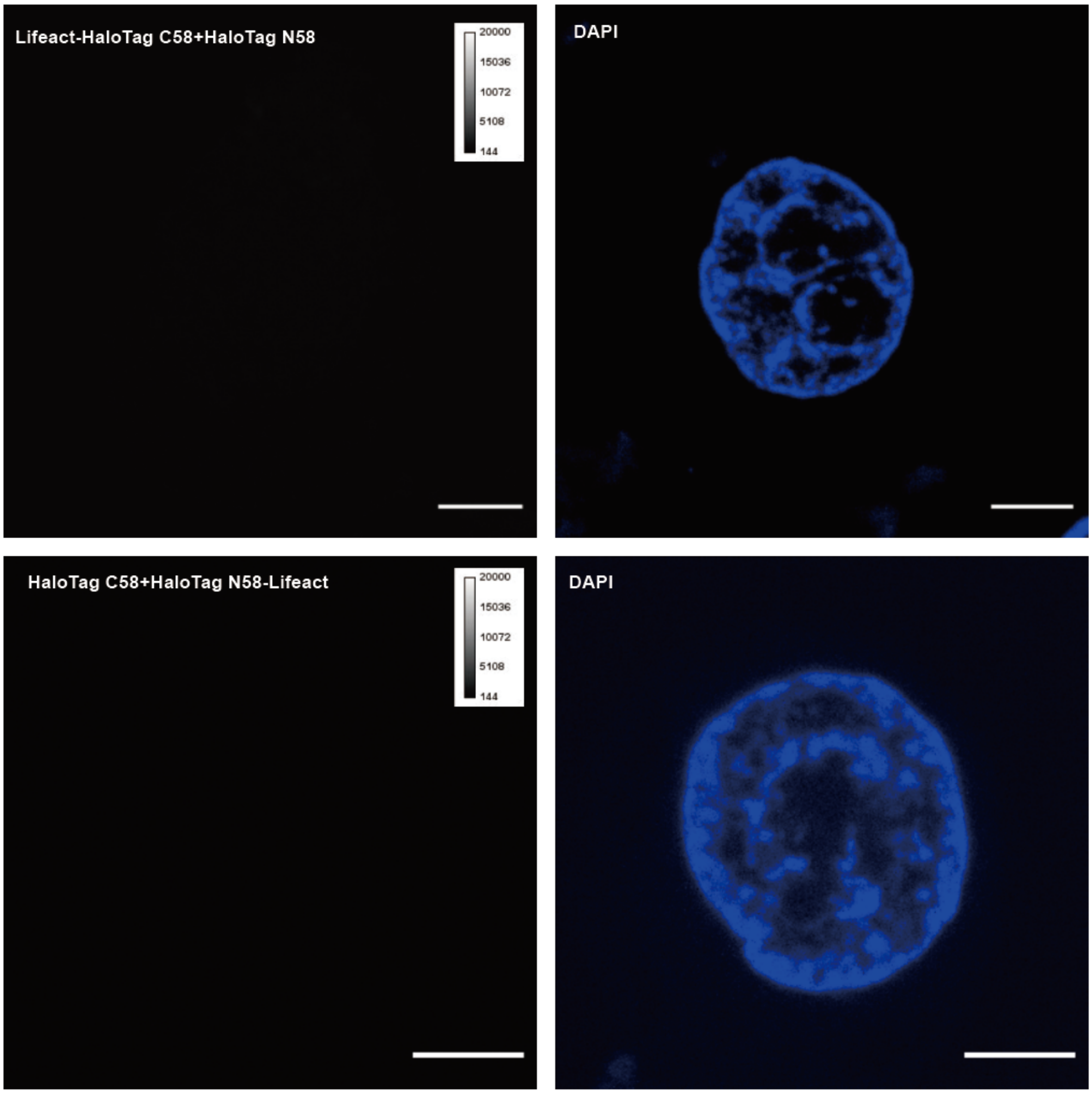

Supplementary Figure 19. Over-expressed TagBiFC probes are invisible.

We have used the cells transfected with Lifeact-HaloTag C58 and HaloTag N58 or HaloTag N58-Lifeact and HaloTag C58 as control experiments to Fig 6b. Both experiments show no actin fluorescence labeling or background signal since split HaloTag half without Lifeact fusion can't bind to actin filament, indicating the background-free imaging of cellular structure using split HaloTag.

Supplementary Table 1. Plasmids used in the manuscript

Plasmid Name	Source
pcDNA3.1(+)-β-Fos-GS-HaloTag C19	This paper
pcDNA3.1(+)-β-Fos-GS-HaloTag C33	This paper
pcDNA3.1(+)-β-Fos-GS-HaloTag C48	This paper
pcDNA3.1(+)-β-Fos-GS-HaloTag C58	This paper
pcDNA3.1(+)-β-Fos-GS-HaloTag C78	This paper
pcDNA3.1(+)-β-Fos-GS-HaloTag C98	This paper
pcDNA3.1(+)-β-Fos-GS-HaloTag C121	This paper
pcDNA3.1(+)-β-Fos-GS-HaloTag C141	This paper
pcDNA3.1(+)-β-Fos-GS-HaloTag C156	This paper
pcDNA3.1(+)-β-Fos-GS-HaloTag C166	This paper
pcDNA3.1(+)-β-Fos-GS-HaloTag C180	This paper
pcDNA3.1(+)-β-Fos-GS-HaloTag C207	This paper
pcDNA3.1(+)-β-Fos-GS-HaloTag C234	This paper
pcDNA3.1(+)-β-Fos-GS-HaloTag C244	This paper
pcDNA3.1(+)-β-Fos-GS-HaloTag C261	This paper
pcDNA3.1(+)-β-Fos-GS-HaloTag C269	This paper
pcDNA3.1(+)-β-Fos-GS-HaloTag C278	This paper
pcDNA3.1(+)-HaloTag N19-GS-β-Jun	This paper
pcDNA3.1(+)-HaloTag N33-GS-β-Jun	This paper
pcDNA3.1(+)-HaloTag N48-GS-β-Jun	This paper
pcDNA3.1(+)-HaloTag N58-GS-β-Jun	This paper
pcDNA3.1(+)-HaloTag N78-GS-β-Jun	This paper
pcDNA3.1(+)-HaloTag N98-GS-β-Jun	This paper
pcDNA3.1(+)-HaloTag N121-GS-β-Jun	This paper
pcDNA3.1(+)-HaloTag N141-GS-β-Jun	This paper
pcDNA3.1(+)-HaloTag N156-GS-β-Jun	This paper
pcDNA3.1(+)-HaloTag N166-GS-β-Jun	This paper
pcDNA3.1(+)-HaloTag N180-GS-β-Jun	This paper
pcDNA3.1(+)-HaloTag N207-GS-β-Jun	This paper
pcDNA3.1(+)-HaloTag N234-GS-β-Jun	This paper
pcDNA3.1(+)-HaloTag N244-GS-β-Jun	This paper
pcDNA3.1(+)-HaloTag N261-GS-β-Jun	This paper
pcDNA3.1(+)-HaloTag N269-GS-β-Jun	This paper
pcDNA3.1(+)-HaloTag N278-GS-β-Jun	This paper
pcDNA3.1(+)-HaloTag N278-GS-β-Jun	This paper
pcDNA3.1(+)-β-Fos (ΔZip)-GS-HaloTag C19	This paper

pcDNA3.1(+)-β-Fos (ΔZip)-GS-HaloTag C33	This paper
pcDNA3.1(+)-β-Fos (ΔZip)-GS-HaloTag C48	This paper
pcDNA3.1(+)-β-Fos (ΔZip)-GS-HaloTag C58	This paper
pcDNA3.1(+)-β-Fos (ΔZip)-GS-HaloTag C78	This paper
pcDNA3.1(+)-β-Fos (ΔZip)-GS-HaloTag C98	This paper
pcDNA3.1(+)-β-Fos (ΔZip)-GS-HaloTag C121	This paper
pcDNA3.1(+)-β-Fos (ΔZip)-GS-HaloTag C141	This paper
pcDNA3.1(+)-β-Fos (ΔZip)-GS-HaloTag C156	This paper
pcDNA3.1(+)-β-Fos (ΔZip)-GS-HaloTag C166	This paper
pcDNA3.1(+)-β-Fos (ΔZip)-GS-HaloTag C180	This paper
pcDNA3.1(+)-β-Fos (ΔZip)-GS-HaloTag C207	This paper
pcDNA3.1(+)-β-Fos (ΔZip)-GS-HaloTag C234	This paper
pcDNA3.1(+)-β-Fos (ΔZip)-GS-HaloTag C244	This paper
pcDNA3.1(+)-β-Fos (ΔZip)-GS-HaloTag C261	This paper
pcDNA3.1(+)-β-Fos (ΔZip)-GS-HaloTag C269	This paper
pcDNA3.1(+)-β-Fos (ΔZip)-GS-HaloTag C278	This paper
pcDNA3.1(+)-c-Fos-GS-HaloTag C58	This paper
pcDNA3.1(+)-HaloTag N58-GS-c-Jun	This paper
pcDNA3.1(+)-FKBP-GS-HaloTag C58	This paper
pcDNA3.1(+)-HaloTag N58-GS-FRB	This paper
pcDNA3.1(+)-FKBP-GS-HaloTag C261	This paper
pcDNA3.1(+)-HaloTag C261-GS-FRB	This paper
pcDNA3.1(+)-c-Fos-GS-mMaple3 C174	This paper
pcDNA3.1(+)-mMaple3 N173-GS-c-Jun	This paper
pcDNA3.1(+)-tdPCP-GS-HaloTag C58	This paper
pcDNA3.1(+)-HaloTag N58-GS-tdMCP	This paper
pcDNA3.1(+)-Lifeact-GS-HaloTag C58	This paper
pcDNA3.1(+)-HaloTag N58-GS-lifeact	This paper
pcDNA3.1(+)-H2A-GS-HaloTag C58	This paper
pcDNA3.1(+)-HaloTag N58-GS-H3	This paper
pcDNA3.1(+)-HaloTag N58-GS-H2A.Z	This paper
pcDNA3.1(+)-CFP-1XMS2-PP7	This paper
pB-TRE-1XMS2-PP7-EF1a-rtTA	This paper
pcDNA3.1(+)-Flag-c-Fos-HaloTag	This paper
pcDNA3.1(+)-Flag-c-Fos-HaloTag C58	This paper
pcDNA3.1(+)-Flag-HaloTag N 58-c-Fos	This paper
phage-cmv-cfp-24xpp7	Wu et al Biophys J. 2012 Jun 20;102(12):2936-44. Epub 2012 Jun 19

Protein sequence

HaloTag N58:

MGSEIGTGFPFDPHYVEVLGERMHYVDVGPRDGTPVLFLHGNPTSSYVWRNIIPHVAP

HaloTag C58:

THRCIAPDLIGMGKSDKPDLGYFFDDHVRFMDAFIEALGLEEVVLVIHDWGSALGFHWA

KRNPERVKGIAFMEFIRPIPTWDEWPEFARETFQAFRTTDVGRKLIIDQNVFIEGTLPMGV

VRPLTEVEMDHYREPFLNPVDREPLWRFPNELPIAGEPANIVALVEEYMDWLHQSPVPKL

LFWGTPGVLIPPAEAARLAKSLPNCKAVDIGPGLNLLQEDNPDLIGSEIARWLSTLEISG

Supplementary Table 2. FISH probes for CFP

Probe Name	Probe Sequence (5'-3')
Cy5-Primary	Cy5-cgagctcggtacccgggtcgCCC
CFP FISH1	GGGcgacccgggtaccgagctcgtcctcgcccttgctcaccat
CFP FISH2	GGGcgacccgggtaccgagctcgtgggcaccaccccggtgaac
CFP FISH3	GGGcgacccgggtaccgagctcgtcgccgtccagctcgaccag
CFP FISH4	GGGcgacccgggtaccgagctcggctgaacttgtggccgttta
CFP FISH5	GGGcgacccgggtaccgagctcgcgccctcgccctcgccggac
CFP FISH6	GGGcgacccgggtaccgagctcggtcagcttgccgtaggtggc
CFP FISH7	GGGcgacccgggtaccgagctcgggtggtgcagatgaacttca
CFP FISH8	GGGcgacccgggtaccgagctcggccagggcacgggcagcttg
CFP FISH9	GGGcgacccgggtaccgagctcgcaggtcagggtggtcacgag
CFP FISH10	GGGcgacccgggtaccgagctcgtagcggctgaagcactgcac
CFP FISH11	GGGcgacccgggtaccgagctcggtgctgcttcatgtggtcgg
CFP FISH12	GGGcgacccgggtaccgagctcggcatggcggacttgaagaag
CFP FISH13	GGGcgacccgggtaccgagctcgcgctcctggacgtagccttc
CFP FISH14	GGGcgacccgggtaccgagctcggtcgtccttgaagaagatgg
CFP FISH15	GGGcgacccgggtaccgagctcgcggcgcgggtcttgtagttg
CFP FISH16	GGGcgacccgggtaccgagctcggtgtcgccctcgaacttcac
CFP FISH17	GGGcgacccgggtaccgagctcgtcagctcgatgcggttcacc
CFP FISH18	GGGcgacccgggtaccgagctcgtcctccttgaagtcgatgcc
CFP FISH19	GGGcgacccgggtaccgagctcgcttgtgccccaggatgttgc
CFP FISH20	GGGcgacccgggtaccgagctcgggctgatgtagttgtactcc
CFP FISH21	GGGcgacccgggtaccgagctcgtcggcggtgatatagacgtt
CFP FISH22	GGGcgacccgggtaccgagctcgcttgatgccgttcttctgct
CFP FISH23	GGGcgacccgggtaccgagctcgtgtggcggatcttgaagttg
CFP FISH24	GGGcgacccgggtaccgagctcgtgcacgctgccgtcctcgat
CFP FISH25	GGGcgacccgggtaccgagctcgtgttctgctggtagtggtcg
CFP FISH26	GGGcgacccgggtaccgagctcgcacggggccgtcgccgatgg
CFP FISH27	GGGcgacccgggtaccgagctcgctcaggtagtggttgtcggg
CFP FISH28	GGGcgacccgggtaccgagctcgctttgctcagggcggactgg
CFP FISH29	GGGcgacccgggtaccgagctcgtgatcgcgcttctcgttggg

CFP FISH30	GGGcgacccgggtaccgagctcgcacgaactccagcaggacca
CFP FISH31	GGGcgacccgggtaccgagctcgcgagagtgatcccggcggcg
CFP FISH32	GGGcgacccgggtaccgagctcgtacttgtacagctcgtccat
