# Supplementary material for: TagBiFC technique allows long-term single-molecule tracking of protein-protein interactions in living cells
Source: Commun Biol. 2021 Mar 19;4:378. doi: 10.1038/s42003-021-01896-7 (PMC7979928; doi:10.1038/s42003-021-01896-7)
Supplement: Supplementary file 2 — Description of Supplementary Files [file 42003_2021_1896_MOESM2_ESM.pdf]

Description of Additional Supplementary Files

File name: Supplementary Data 1

Description: The Source data underlying Figure 1c and Figure 1g.

File name: Supplementary Data 2

Description: The Source data underlying Figure 2b-d.

File name: Supplementary Data 3

Description: The Source data underlying Figure 3a-j.

File name: Supplementary Data 4

Description: The Source data underlying Figure 4c-k.

File name: Supplementary Data 5

Description: The source data underlying Figure 5d and Figure 5j.
