# Supplementary material for: TagBiFC technique allows long-term single-molecule tracking of protein-protein interactions in living cells
Source: Commun Biol. 2021 Mar 19;4:378. doi: 10.1038/s42003-021-01896-7 (PMC7979928; doi:10.1038/s42003-021-01896-7)
Supplement: Supplementary file 8 — Reporting Summary [file 42003_2021_1896_MOESM8_ESM.pdf]

Corresponding author(s): Yujie Sun

Last updated by author(s): Shipeng Shao

Reporting Summary

Nature Research wishes to improve the reproducibility of the work that we publish. This form provides structure for consistency and transparency in reporting. For further information on Nature Research policies, see our Editorial Policies and the Editorial Policy Checklist.

Statistics

For all statistical analyses, confirm that the following items are present in the figure legend, table legend, main text, or Methods section.

n/a	Confirmed	
☐	☒	The exact sample size (*n*) for each experimental group/condition, given as a discrete number and unit of measurement
☐	☒	A statement on whether measurements were taken from distinct samples or whether the same sample was measured repeatedly
☐	☒	The statistical test(s) used AND whether they are one- or two-sided<br>*Only common tests should be described solely by name; describe more complex techniques in the Methods section.*
☒	☐	A description of all covariates tested
☒	☐	A description of any assumptions or corrections, such as tests of normality and adjustment for multiple comparisons
☐	☒	A full description of the statistical parameters including central tendency (e.g. means) or other basic estimates (e.g. regression coefficient) AND variation (e.g. standard deviation) or associated estimates of uncertainty (e.g. confidence intervals)
☐	☒	For null hypothesis testing, the test statistic (e.g. *F*, *t*, *r*) with confidence intervals, effect sizes, degrees of freedom and *P* value noted<br>*Give P values as exact values whenever suitable.*
☒	☐	For Bayesian analysis, information on the choice of priors and Markov chain Monte Carlo settings
☒	☐	For hierarchical and complex designs, identification of the appropriate level for tests and full reporting of outcomes
☒	☐	Estimates of effect sizes (e.g. Cohen's *d*, Pearson's *r*), indicating how they were calculated

Our web collection on statistics for biologists contains articles on many of the points above.

Software and code

Policy information about availability of computer code

Data collection	All data are collected on a custom Olympus IX83 inverted microscope equipped with a 100X UPlanSApo, N.A. = 1.49, oil-immersion phase objective and Andor iXon Ultra EMCCD.
Data analysis	RStudio, MatLab and Microsoft Excel.

For manuscripts utilizing custom algorithms or software that are central to the research but not yet described in published literature, software must be made available to editors and reviewers. We strongly encourage code deposition in a community repository (e.g. GitHub). See the Nature Research guidelines for submitting code & software for further information.

Data

Policy information about availability of data

All manuscripts must include a data availability statement. This statement should provide the following information, where applicable:

- Accession codes, unique identifiers, or web links for publicly available datasets
- A list of figures that have associated raw data
- A description of any restrictions on data availability

All data generated and analyzed during the current study are available from the corresponding author on reasonable request.

Field-specific reporting

Life sciences study design

All studies must disclose on these points even when the disclosure is negative.

Sample size	more than 10 cells were used to extract the single molecule dynamics
Data exclusions	no data were excluded in the samples
Replication	Each experiment was repeated 2-3 times
Randomization	n/a
Blinding	n/a

Reporting for specific materials, systems and methods

We require information from authors about some types of materials, experimental systems and methods used in many studies. Here, indicate whether each material, system or method listed is relevant to your study. If you are not sure if a list item applies to your research, read the appropriate section before selecting a response.

Materials & experimental systems

n/a	Involved in the study	
☐	☒	Antibodies
☐	☒	Eukaryotic cell lines
☒	☐	Palaeontology and archaeology
☒	☐	Animals and other organisms
☒	☐	Human research participants
☒	☐	Clinical data
☒	☐	Dual use research of concern

Methods

n/a	Involved in the study	
☒	☐	ChIP-seq
☒	☐	Flow cytometry
☒	☐	MRI-based neuroimaging

Antibodies

Antibodies used	c-Fos (9F6) Rabbit mAb CST#2250, c-Jun (60A8) Rabbit mAb CST#9165, Flag (D6W5B) Rabbit mAb CST#14793, GAPDH Mouse Monoclonal (Proteintech-60004-1-Ig)
Validation	Validation of the all the antibodies we used were described in the manufacturers website

Eukaryotic cell lines

Policy information about cell lines

Cell line source(s)	MDA-MB-231 was a gift from Dr. Wei Guo (Department of Biology, University of Pennsylvania)
Authentication	None of the cell lines were authenticated within 1 year
Mycoplasma contamination	All cell lines were tested negative for mycoplasma contamination
Commonly misidentified lines (See ICLAC register)	No misidentified cell lines
